# Science-based suggestions to save the world’s rarest primate species *Nomascus hainanus*

**DOI:** 10.1126/sciadv.adv4828

**Published:** 2025-04-11

**Authors:** Xukai Zhong, Xia Huang, Changyue Zhu, Yuxin Wang, Colin A. Chapman, Paul A. Garber, Yuan Chen, Pengfei Fan

**Affiliations:** ^1^ School of Life Sciences, Sun Yat-Sen University, Guangzhou 510275, China.; ^2^ Key Laboratory of Conservation and Application in Biodiversity of South China, School of Life Sciences, Guangzhou University, Guangzhou 510006, China.; ^3^ Biology Department, Vancouver Island University, Nanaimo, British Columbia, V9R 5S5, Canada.; ^4^ Wilson Center,1300 Pennsylvania Avenue NW, Washington, DC 20004, USA.; ^5^ School of Life Sciences, University of KwaZulu-Natal, Scottsville, Pietermaritzburg 3209, South Africa.; ^6^ Shanxi Key Laboratory for Animal Conservation, Northwest University, Xi’an 710127, China.; ^7^ Department of Anthropology and Program in Ecology, Evolution, and Conservation Biology, University of Illinois, Urbana, IL 61801, USA.; ^8^ International Centre of Biodiversity and Primate Conservation, Dali University, Dali, Yunnan, China.; ^9^ School of Ecology, Hainan University, Haikou 570228, China.

## Abstract

Conservation practices for extremely small populations must be grounded in solid science to prevent extinction. Hainan gibbon (*Nomascus hainanus*) is the world’s rarest primate species; however, insufficient data on its habitat suitability and genetic status impede evidence-based decisions for habitat restoration. Here, we conducted a comprehensive analysis of Hainan gibbons’ energy intake and expenditure, reproductive parameters, and genetic diversity based on field research (March 2021 to December 2022) and long-term historical data (2003 to 2024). By comparing our results with those of captive gibbons and other free-feeding captive primates, we found that Hainan gibbons can obtain sufficient energy for growth and reproduction in their existing habitats. Furthermore, we identified an additional D-loop haplotype indicating that the current population is more genetically diverse than previously thought. However, recently formed adult male-female pairs are increasingly related, signaling a high risk for inbreeding within this small population. Based on these findings, we highlight an urgent need to expand available habitat by building corridors.

## Teaser

The recovery of Hainan gibbons must be grounded in solid science.

## INTRODUCTION

Saving species with extremely small populations is challenging (*1*). The successful stories [e.g., the Crested Ibis *Nipponia nippon* (*2*, *3*); California condor *Gymnogyps californianus* (*4*)] have been notably few, particularly when it comes to species with long life history and low reproductive output, such as large mammals [e.g., the Yangtze River dolphin *Lipotes vexillifer* (*5*); the Northern White rhinoceros *Ceratotherium simum cottoni* (*6*)]. Once populations decline below a critical threshold, a species enters the extinction vortex, where demographic, environmental, and genetic stochasticity consistently render it vulnerable to extinction (*7*, *8*). The deteriorated environment (e.g., nutritional deprivation), genetic issues (e.g., inbreeding depression, genetic bottleneck), or stochastic events (e.g., extreme weather) can precipitate swift population extinction or impede population recovery (*9*, *10*). Under these circumstances, human interventions (e.g., habitat restoration and captive breeding) become imperative (*11*, *12*). However, interventions must be grounded in solid science to ensure that financial resources are not squandered on endeavors that will not lead to success. For example, habitat restoration in small disparate areas for the Iberian lynx (*Lynx pardinus*), a species with large home range requirements, contributed minimally to their population recovery (*13*). Sometimes, interventions without a scientific basis can even be detrimental. For example, food supplementary to the critically endangered Kakapo (*Strigops habropitilus*) without considering the nutritional composition of its diet led to obesity issues within the population (*14*, *15*).

The Hainan gibbon (*Nomascus hainanus*) is the world’s rarest primate species and one of the rarest mammals (*16*). This species is endemic to the Hainan Island, China and was once widespread throughout the island (*17*). However, since the 1950s, there has been a marked population decline due to high levels of poaching and rapid habitat loss induced by large-scale logging and the establishment of pine or rubber plantations (*18*). Their population decreased to less than 40 individuals in the 1970s, with only seven and eight individuals left in Bawangling (*19*). Other populations subsequently went extinct, and only the Bawangling population survived and has since fluctuated at an extremely low level (*17*, *20*). In October 2003, an international collaborative survey covered all potential habitats and only detected two social groups and two solitary individuals, totaling 13 individuals with three breeding females (*21*, *22*), rending it the primate species most likely to go extinct (*23*). Subsequently, this small population received extensive conservation attention, leading to the launch of the first Hainan Gibbon Conservation Action Plan in 2005 (*22*). Conservation actions included periodic population monitoring, intensive patrolling, and lowland forest restoration (*24*). Hunting was completely controlled, and the population gradually increased to 42 individuals by 2024, consisting of seven groups with 12 breeding females (*25*). In October 2020, with the establishment of the Hainan Tropical Rainforest National Park (HNTRNP), Hainan gibbon was designated as flagship species for the park (*16*). Consequently, it became the “giant panda to Hainan” and has attracted more and more conservation resources (*26*, *27*). Several hundreds of millions of Renminbi¥ have been invested or are planned for the protection of this species and its habitat.

Poor habitat quality and low genetic diversity have long been suggested to hinder the recovery of the Hainan gibbon population (*20*). From the 1950s to the 1990s, extensive deforestation occurred in the lowland tropical forests of Hainan Island, resulting in the remnant gibbon population persisting solely in high-altitude forests (*22*). These gibbon groups were suggested to occupy an unusually large home range (400 to 900 ha) and use a high proportion of secondary forest, leading to a belief that Hainan gibbons live in a poor habitat that could not meet adequately their energetic and nutritional needs (*28*). Thus, suggestion that enhancing existing habitat quality would be the most appropriate conservation action has been made (*28*, *29*). However, recent research has revealed that Hainan gibbons occupy a home range (100 to 200 ha) comparable to that of other *Nomascus* gibbon species (*30*, *31*) and have a fruit-rich diet (*32*). They suggested that it is not necessary to improve quality of the current habitat; instead, it would be more beneficial to expand the available habitat (*31*, *32*). However, a fruit-rich diet does not necessarily correlate with good nutritional quality of Hainan gibbons’ habitat or the fulfillment of their energy needs. In this regard, measures of energy acquisition and reproductive dynamics can serve as reasonable indicators of habitat quality (*33*) and be used to prioritize effective management strategies. Furthermore, the genetic diversity of the Hainan gibbon population was found significantly lower than historic levels (*34*, *35*). Although the Hainan gibbon population has increased rapidly in the past two decades, its low genetic diversity poses a high risk of inbreeding, which may reduce reproductive performance and increase susceptibility to disease, consequently impeding the recovery of the small population (*11*, *36*). Establishing a captive breeding population and adopting artificially assisted reproductive techniques have been proposed as an effective tool to increase reproductive rate and reduce the risk of the population collapse in response to extreme weather events, unpredictable natural disasters, or disease.

These different proposed actions each necessitate substantial allocation of resources and will have profound influence on the conservation of Hainan gibbon. Thus, a comprehensive evaluation of habitat quality and genetic diversity is needed to prioritize effective conservation strategies. To accomplish this, we (i) habituated two Hainan gibbon groups with different coverage of secondary forest within their home range [20.736% versus 62.16%; (*37*)] and conducted a year-round observation of their feeding behavior (17,249 feeding records during 1344 hours over 179 days). We collected data on feeding rates and analyzed the macronutrient composition of their foods. We use these data to present the estimates of energy intake and expenditure in Hainan gibbons. This is also the only available data on energy intake for any wild gibbon population. We then estimated the energy intake of four well-fed captive populations of yellow-cheeked gibbons (*Nomascus gabriellae*), a close relative of Hainan gibbons, and compared the results for our wild gibbons with these captive gibbon populations and with other captive free-feeding primates to assess whether Hainan gibbons can obtain sufficient energy from their current habitats. (ii) We also compiled a detailed dataset spanning over 20 years of female reproduction and age at dispersal for Hainan gibbons from the published literatures, long-term field records from the Bawangling Nature Reserve (now a part of the Hainan Tropical Forest National Park), and our own field data. We compared interbirth interval (IBI) and male dispersal age (MDA) of Hainan gibbons with those of other gibbon species to assess how well the population is reproducing in their current habitats. (iii) We compiled a genetic dataset including nearly all adult members (18 of 21) of the current Hainan gibbon population to evaluate its genetic diversity and potential inbreeding. Using these exhaustive datasets, we evaluate habitat quality and genetic issues of this tiny population to provide a comprehensive understanding of the Hainan gibbon’s survival status. This allows us to make a solid science–based evaluation of different conservation strategies for this critically endangered ape species.

## RESULTS

### Daily macronutrient and energy intake

The annual average daily dry matter intake (DMI) of adult Hainan gibbons was estimated to be 342.47 g·day^−1^ {95% confidence interval (CI): [287.90, 397.04]{, accompanied by an annual average daily energy intake (DEI) of 935.57 kcal·day^−1^ (95% CI: [800.72, 1,070.43]). The DEI comprised a protein energy intake of 86.06 kcal·day^−1^ (95% CI: [71.00, 101.12]), a fat energy intake of 117.77 kcal·day^−1^ (95% CI: [89.80, 145.73]), a nonstructural carbohydrate energy intake of 669.52 kcal·day^−1^ (95% CI: [584.58, 754.47]), and a fiber energy intake of 62.22 kcal·day^−1^ (95% CI: [47.48, 76.97]).

**Fig. 1.**
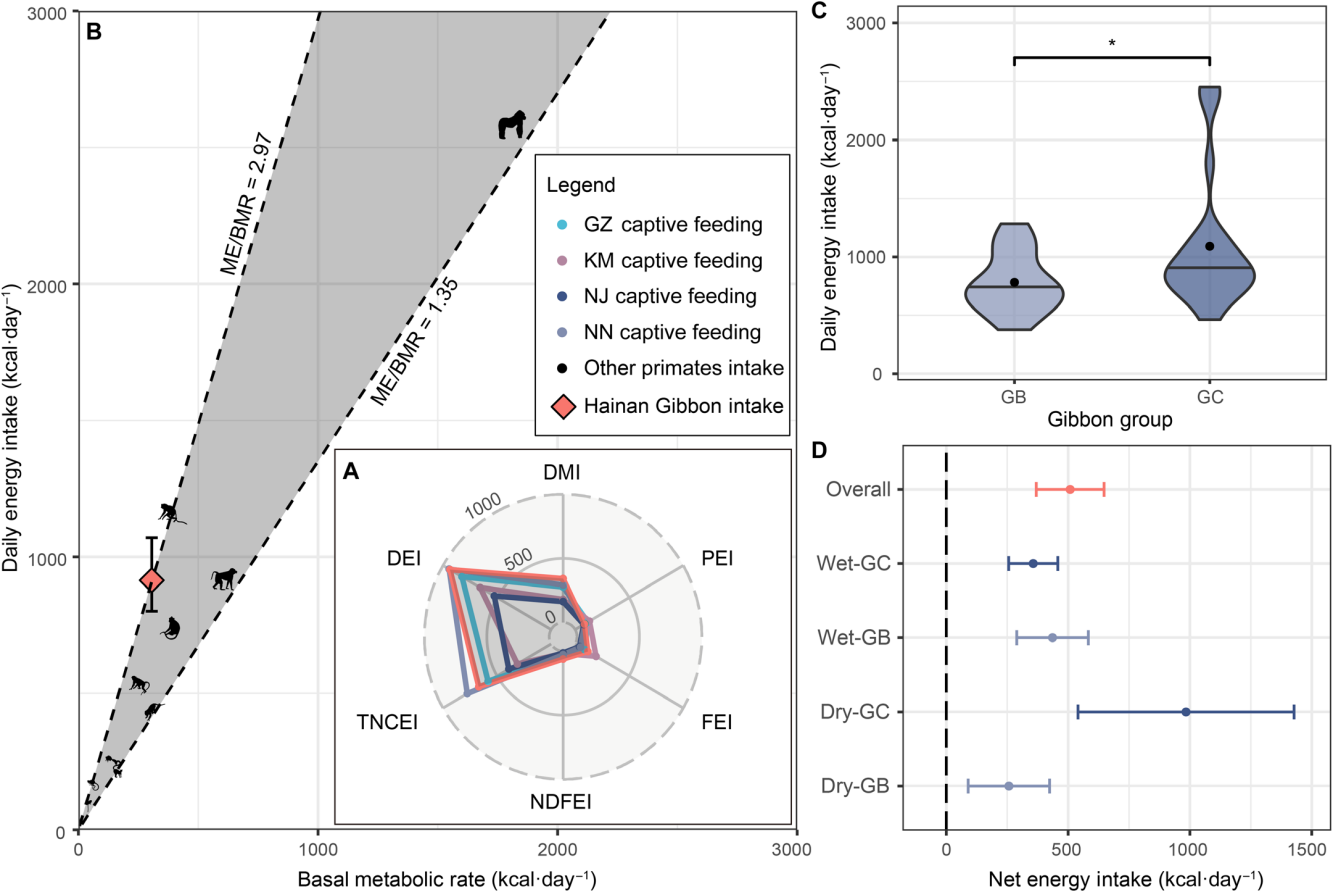
Macronutrient and energy intake of the Hainan gibbon. (**A**) Annual average daily DMI (g·day^−1^), PEI (kcal·day^−1^), FEI (kcal·day^−1^), neutral detergent fiber energy intake (NDFEI; kcal·day^−1^), total nonstructural carbohydrate energy intake (TNCEI; kcal·day^−1^), and DEI (kcal·day^−1^) of wild Hainan gibbons and captive yellow-cheeked gibbons from four zoos (GZ, Guangzhou Zoo; KM, Kunming Zoo; NJ, Nanjing Zoo; NN; Nanning Zoo). (**B**) Average DEI and BMR of wild Hainan gibbons and captive free-feeding primates. The black patterns represent these 11 different primates [BMR from large to small: great ape (Gorilla sp. and *Pongo* sp.), baboon (Papio sp.), rhesus monkey (*Macaca mulatta*), proboscis monkey (*Nasalis larvatus*), howler monkey (Alouatta sp.), long-tailed Macaque (*Macaca fascicularis*), lemur (Lemur sp.), Aye-aye (*Daubentonia madagascariensis*), squirrel monkey (Saimiri sp.), tamarin (Saguinus sp.), and marmoset (*Callithrix* sp. and *Saguinus* sp.); table S1]. Their DEI and BMR were obtained from the National Research Council (2003) (*40*). The red angular shape represents wild Hainan gibbons, and the error bar represents the 95% CI for the DEI found in this study. The two dotted diagonal lines represent the range encompassing the lowest and highest ratios of DEI to BMR among the captive free-feeding primates. (**C**) Comparison of DEI between wild Hainan gibbon group GB and GC. “*” represents a significant difference in the DEI between the two gibbon groups. (**D**) NEI of the two wild Hainan gibbon groups in the wet and dry season. The error bar represents the 95% CI for the NEI.

The average dry matter provided to captive yellow-cheeked gibbons in the four zoos was 228.27 g·day^−1^ (ranging from 162.93 to 293.54), which corresponded to an average metabolic energy intake of 737.98 kcal·day^−1^ (ranging from 526.09 to 943.89). The metabolic energy consisted of a protein energy of 104.79 kcal·day^−1^ (ranging from 79.79 to 132.04), a fat energy of 93.53 kcal·day^−1^ (ranging from 40.66 to 194.08), a nonstructural carbohydrate energy of 515.73 kcal·day^−1^ (ranging from 312.38 to 775.94), and a fiber energy of 23.93 kcal·day^−1^ (ranging from 14.24 to 35.32). In comparison, the energy and macronutrient intakes of wild Hainan gibbons were either higher or fell within the range of those provided to captive yellow-cheeked gibbons in zoos (Fig. 1A).

Based on an average body weight (BW) of six Hainan gibbons from the published literature (*38*, *39*), we estimated average basal metabolic rate (BMR) of adult Hainan gibbons to be 306.07 kcal·day^−1^. The average ratio of DEI to BMR for wild Hainan gibbons was estimated to be 3.06 (95% CI: [2.62, 3.50]; Fig. 1B). The average ratio of DEI to BMR for 11 species of captive free-feeding primates (*40*), which were provided with abundant food and had free access to feeding, was 1.94, with a range from 1.35 to 2.97 (Fig. 1B). The higher DEI-to-BMR ratio observed in wild Hainan gibbons suggests that they can meet their daily energy requirements within the habitats they currently occupy.

The DEI of adult Hainan gibbons varied among months. However, we used a multivariable linear regression model with DEI as dependent variable and group, sex, and season as independent variables (*N* = 48) and found no significant differences in DEI between sexes (|Estimate| = 0.081, SE = 0.111, *P* = 0.469) or seasons (|Estimate| = 0.037, SE = 0.111, *P* = 0.741). The DEI of the two Hainan gibbon groups were found to differ (|Estimate| = 0.281, SE = 0.111, *P* = 0.014), with group GC having a higher DEI than group GB’s (mean ± SD, GB: 781.81 ± 254.26 kcal·day^−1^, GC: 1089.33 ± 571.63 kcal·day^−1^; Fig. 1C).

The annual average daily energy expenditure (DEE) of adult Hainan gibbons was estimated to be 427.14 kcal·day^−1^ (95% CI: [418.03, 436.24]), while the annual average net energy intake (NEI) was estimated to be 508.44 kcal·day^−1^ (95% CI: [369.29, 647.59]). Similar to the DEI, a multivariable linear regression model (*N* = 48) also found differences between groups (|Estimate| = 0.573, SE = 0.211, *P* = 0.009), but no differences were found between sexes (|Estimate| = 0.238, SE = 0.211, *P* = 0.267) or seasons (|Estimate| = 0.057, SE = 0.212, *P* = 0.788). Group GC had a higher NEI than group GB (GB: 346.51 ± 258.84 kcal·day^−1^, GC: 670.36 ± 589.55 kcal·day^−1^). The NEI values of both Hainan gibbon groups during the dry and wet seasons significantly surpassed 0 (Fig. 1D).

### Female IBI and MDA

**Fig. 2.**
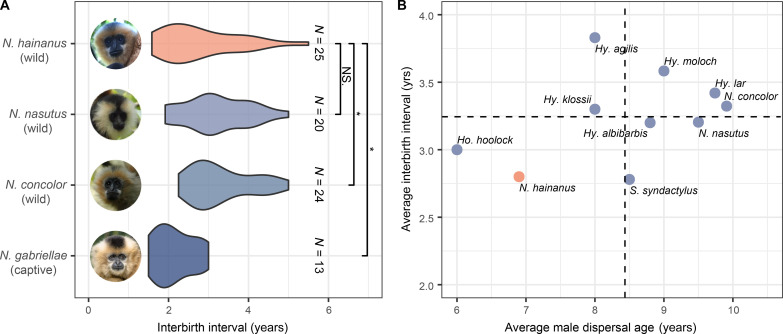
Comparison of IBIs and MDA between the Hainan gibbon and other gibbon species. (**A**) Comparison of female IBIs between wild Hainan gibbon (2003–2024) and captive yellow-cheeked gibbons (2003–2018) (*81*), as well as two other species of wild *Nomascus* gibbons (the Cao vit gibbon from 2008 to 2024 and the western black-crested gibbon from 2003 to 2024). *N* represents the number of female interbirth events. “*” represents a significant difference in the IBI between species, whereas “NS.” indicates no significant difference. Photo credit of *N. hainanus*: Xukai Zhong, Sun Yat-Sen University. Photo credit of *N. nasutus* and *N. concolor*: Chao Zhao, Cloud Mountain Conservation. Photo credit of *N. gabriellae*: Dirk Rabe, Public Domain. (**B**) Average IBI and average MDA among 10 gibbon species (table S2). The horizontal and vertical dotted lines depict the average values of that variable across all gibbon species in the diagram.

We present data on Hainan gibbon reproductive performance and use this as a proxy of habitat quality. We assume that a primate group that is approaching maximum reproductive output inhabits a home range that can provide for its nutritional needs year-round. The IBI of our population of wild Hainan gibbons was found to be 2.80 ± 0.98 (Mean ± SD) years (*N* = 25). Two other species of Chinese *Nomascus* gibbons, the Cao vit gibbon (*Nomascus nasutus*) and the western black-crested gibbon, (*Nomascus concolor*), which have also been monitored for almost two decades through our own fieldwork, exhibited female IBIs of 3.20 ± 0.84 years (*N* = 20) and 3.32 ± 0.77 years (*N* = 24), respectively. We used a linear mixed model (LMM) to test for significant differences in the IBI between the Hainan gibbons and the other three gibbon species (*N* = 82). The IBI was set as the dependent variable, with the gibbon species specified as the fixed effect and the Hainan gibbon serving as the intercept. The gibbon individual was considered a random effect. We found that the IBI of the Hainan gibbon was significantly shorter than that of the Western black-crested gibbon (Estimate = 0.199, SE = 0.077, *P* = 0.012) while not significantly different from that of the Cao vit gibbon (Estimate = 0.154, SE = 0.081, *P* = 0.062) (Fig. 2A). Captive yellow-cheeked gibbons exhibited a significantly shorter IBI of 2.15 ± 0.49 years (*N* = 13) compared to the wild Hainan gibbons (Estimate = −0.234, SE = 0.092, *P* = 0.014) (Fig. 2A). The average IBI of wild populations across 10 gibbon species was 3.25 years, with a range of 2.78 to 3.83. The Hainan gibbon exhibited the second shortest IBI among these species (Fig. 2B).

Given that gibbon groups commonly contain only a single adult male, the dispersal of males from their natal groups is an indicator that the male has reached sexual maturity (*41*). Males across ten gibbon species dispersed from their natal groups at an average age of 8.43 years (ranging from 6.00 to 9.91). Notably, male Hainan gibbons exhibited the second earliest age of dispersal at 6.90 ± 1.14 years (*N* = 5, Fig. 2B).

### Genetic diversity and pairwise relatedness

We collected fresh fecal samples of individuals within the Hainan gibbon population to investigate its genetic diversity and pairwise relatedness. On the basis of genotype data of microsatellite loci and sexual loci, we obtained reliable microsatellite genotypes for 31 Hainan gibbon individuals (male: *N* = 18, female: *N* = 13), including eight individuals from two previous studies (table S3) (*35*, *42*). Among the 10 offspring, eight were males and two were females.

**Fig. 3.**
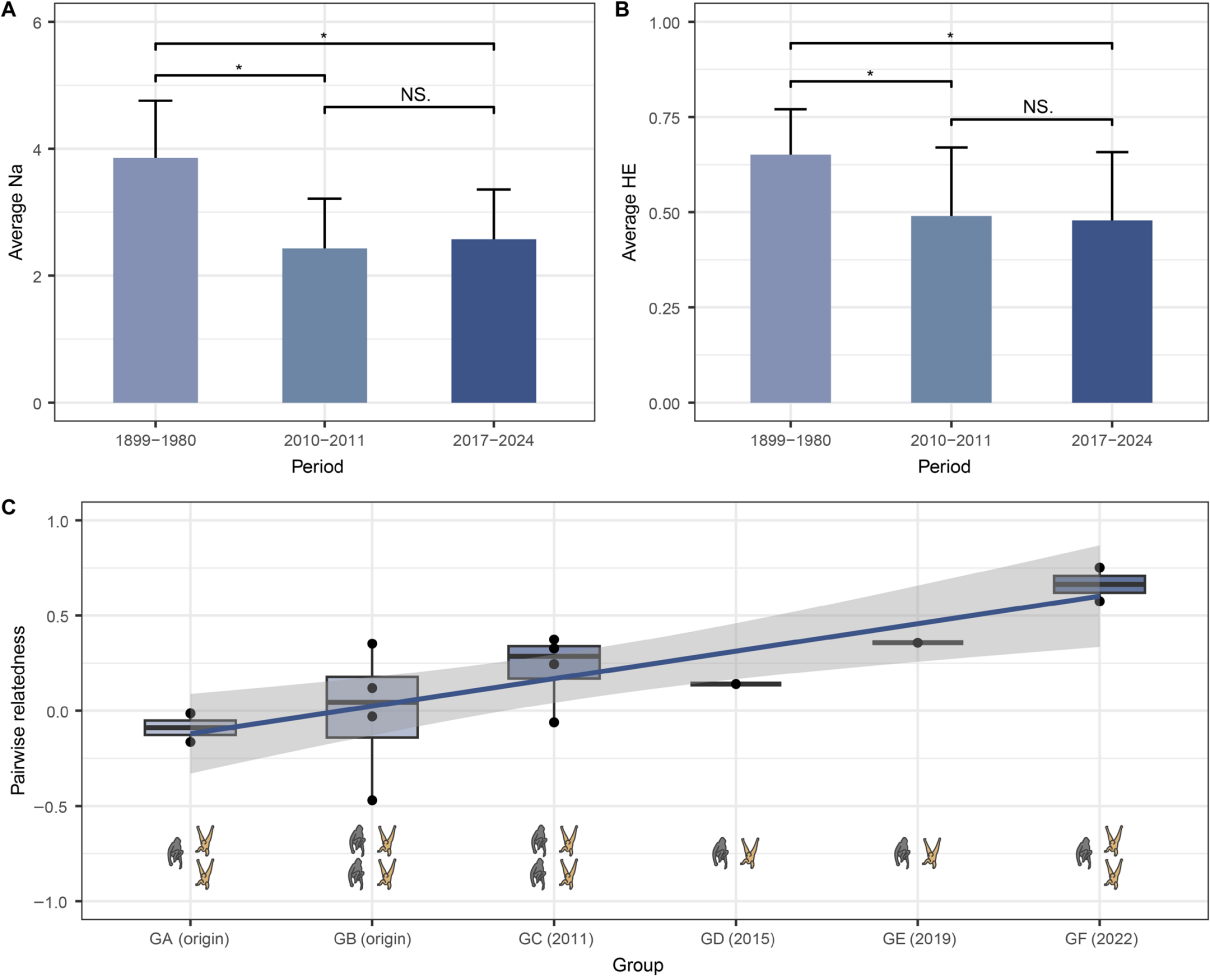
Genetic diversity and inbreeding status of the current wild Hainan gibbon population. The genetic diversity of the current wild Hainan gibbon population (2017–2024) is compared to those of the historical population (1899–1980) and the wild population in the previous decade (2010–2011) as presented in Bryant *et al.* (*34*). (**A**) Comparison of the number of different alleles (Na) between the historical and current population of Hainan gibbons. (**B**) Comparison of the expected heterozygosity (*H*_E_) between the historical and current population of Hainan gibbons. (**C**) Pairwise relatedness between intermating adult males and females in the six extant Hainan gibbon groups. The numbers within parentheses show the formation time of the gibbon groups, with group GA and GB being the two original groups in 2003. The gibbon patterns indicate the number of adult individuals included in the pairwise relatedness analysis for each group. A gray pattern represents a male individual, while a yellow pattern represents a female individual.

The genetic diversity of the current population (2017–2024) was still decreasing. Using the same microsatellite loci, we found that both the average number of different alleles (Na) and expected heterozygosity (*H*_E_) in the current population were significantly lower compared to the historical diversity (1899–1980) reported by Bryant *et al.* (*34*) (*Na*: *N* = 7, *V* = 21, *P* = 0.031; *H*_E_: *N* = 7, V = 28, *P* = 0.016; Fig. 3A). However, Wilcoxon signed-rank test found no significant difference between the current population and population from 2010 to 2011 (Na: *N* = 7, *V* = 0, *P* = 1.000; *H*_E_: *N* = 7, *V* = 18, *P* = 0.578; Fig. 3B). The average inbreeding coefficient (*F*_IS_) was −0.306 based on 10 microsatellite loci. The pairwise relatedness of the current Hainan gibbon population was 0.281 ± 0.318 (means ± SD). We used a linear regression model (*N* = 14) and found that the pairwise relatedness between intermating pairs exhibited a significant positive correlation with the temporal sequence of group formation (Estimate = 0.159, SE = 0.041, *P* = 0.002). The recently formed gibbon group GF showed the highest pairwise relatedness between intermating pairs, reaching an average value of 0.664 (Fig. 3C).

The number of haplotypes present in the current Hainan gibbon population was three (H1: *N* = 6, H2: *N* = 16, H3: *N* = 2), haplotype diversity (Hd) was 0.507, and nucleotide diversity (Pi) was 0.00506. There were two haplotypes in group GA, GB, GC, and GE and one in group GF. Two breeding females had the H1 haplotype. One breeding female had the H3 haplotype. Five breeding females had the H2 haplotype (fig. S1).

## DISCUSSION

Our study provides the estimation of annual DEI for wild Hainan gibbons and to our knowledge is the first quantification of energy intake among any gibbon species. In conjunction with reproductive parameters derived from two decades of monitoring this last remaining Hainan gibbon population, we provide an in-depth understanding on the critically endangered gibbon species’ adaptation to their current habitats. We also conducted a comprehensive reassessment of the overall genetic diversity of Hainan gibbons based on an extensive sampling effort to elucidate the underlying changes in genetic diversity that occurred in conjunction with their steady population growth. Building on these findings, we address existing controversies surrounding conservation strategies and propose specific recommendations as well as potential knowledge gaps for future conservation planning to protect this species.

### Current habitats are not poor

Our estimation of the energy intake and expenditure of wild Hainan gibbons demonstrates that their current habitat adequately fulfills their energy requirements. Compared to well-fed captive *Nomascus* gibbons, wild Hainan gibbons exhibited higher DEI, and their macronutrient intake fell within the corresponding range of captive gibbons (Fig. 1A). Moreover, the ratio of DEI to BMR in Hainan gibbons surpassed that observed in most captive free-feeding primates provided with an abundant quantity of food and allowed unrestricted access to feeding (Fig. 1B). Certainly, wild gibbons, especially those inhabiting high elevations, are expected to experience higher energy costs than captive gibbons, due in part to the fact that wild gibbons travel 1 to 2 km each day and require additional energy for potential cold stress at night (*43*, *44*). However, studies of primate exercise physiology indicate that the ecological cost of transport, defined as the “percentage of the total DEE accounted for by locomotion”, is expected to represent only 1 to 5% of an individuals’ total daily energy requirements (*45*). Gibbons can also adjust their thermoregulatory behavior by sleeping at lower elevations and closer to potential feeding trees, thereby compensating for any additional nighttime energy costs (*44*). Our findings, therefore, suggest that the Hainan gibbons can acquire sufficient energy to meet their needs within their current habitats. In addition, their *NEI* remained positive across seasons, indicating no discernible seasonal energy bottleneck (Fig. 1D).

In primates, reproductive rate typically exhibits a positive correlation with diet quality (*46*, *47*). The IBI of wild Hainan gibbons was comparable and, in many cases, shorter than that reported in other wild gibbon populations including gibbons that live in tropical forests (Fig. 2, A and B). Favorable nutritional conditions can lead to earlier sexual maturity in gibbons, as observed in captive populations (*48*, *49*). Hainan gibbons have one of the shortest IBI and the earliest male maturation age of any gibbon species (Fig. 2B), indicating a robust reproductive capacity of the current population inhabiting their current habitat. The Hainan gibbon diet includes 85% fruit (*32*), the nonstructural carbohydrates of which accounts for 70% of energy intake. A females’ shorter breeding interval and a males’ earlier age at maturation, suggest that Hainan gibbons remain well suited to their current environment. This argues against the suggestion of poor habitat quality for the Hainan gibbon (*28*, *29*) and suggests that future conservation efforts should prioritize expanding available habitat over restoring existing habitat.

Secondary forests are often perceived as suboptimal habitats for many animals (*50*, *51*). In terms of the two Hainan gibbon group studied, GC’s habitat had more secondary forest (62% compared to that of GB with 21%) (*37*). However, the DEI and NEI of group GC were higher than those of the group GB throughout the year (Fig. 1C). The main disparity between groups was primarily manifested during the dry season (Fig. 1D) when gibbons rely more on figs due to the low availability of berry fruits (*32*). This may be related to the greater abundance of figs, an important fallback food for gibbons (*52*, *53*), within the habitat of group GC (*32*). Fig trees are unsuitability for wood production, so their abundance has likely been influenced by selective logging practices. Our results indicate that in the case of Hainan gibbons, extensive secondary forest habitats do not necessarily indicate poorer habitats (*54*). Thus, relying solely on forest type for the classification of habitat quality presents limitation. Future assessment and restoration planning for the habitat of Hainan gibbons should be based on a comprehensive investigation of its feeding ecology and quantitative data on the availability and distribution of their food trees.

### Limited genetic diversity will require conservation action

Consistent with the findings of previous studies (*34*, *35*), the genetic diversity of Hainan gibbons remains alarmingly low. Despite their increasing population, the genetic diversity is lower than historical levels (Fig. 3A). Furthermore, among the seven microsatellite loci compared, the *H*_E_ of four loci was found to have declined over the past decade (Fig. 3B). Previous research indicated that there were only two D-loop haplotypes in the population (*55*). We found an additional D-loop haplotype with a polymorphic site differing from that reported by Guo *et al.* (*55*). Although this may be due to limited previous sampling effort, it signifies positive news for the future recovery of genetic diversity in wild Hainan gibbons.

The risk of inbreeding poses a substantial obstacle to the recovery of small populations (*56*). Previous studies have proposed a potential risk of inbreeding among Hainan gibbons, as adult male-female pairs in the later-formed group GC are more closely related compared to those in group GA and GB (*34*, *35*). In this study, the negative average *F*_IS_ value suggests no pronounced inbreeding within the current Hainan gibbon population as a whole. However, our larger sample size including the three most recently formed gibbon groups (GD, GE, and GF) confirmed a significant trend that the recently formed adult male-female pairs are increasingly related (Fig. 3C), indicating an elevated susceptibility to consanguineous mating. Furthermore, the pairwise relatedness between intermating adult males and females in a Hainan gibbon group (GF) exceeded 0.5, revealing that inbreeding has occurred. Gibbons typically establish enduring pair bonds, and the negative effects of inbreeding can become apparent after a few generations. The occurrence of inbreeding may result from the proximity of Hainan gibbons’ current habitat to saturation levels (*20*, *37*), coupled with the overall limited genetic diversity. The limited habitat availability restricts long distance dispersal, thereby severely constraining mate selection. This suggests that a conservation strategy designed to rapidly expand their habitat, enabling migrating gibbons to freely move and select among more mates and promoting gene flow would be advantageous.

### Building corridors to rapidly expand available habitats

**Fig. 4.**
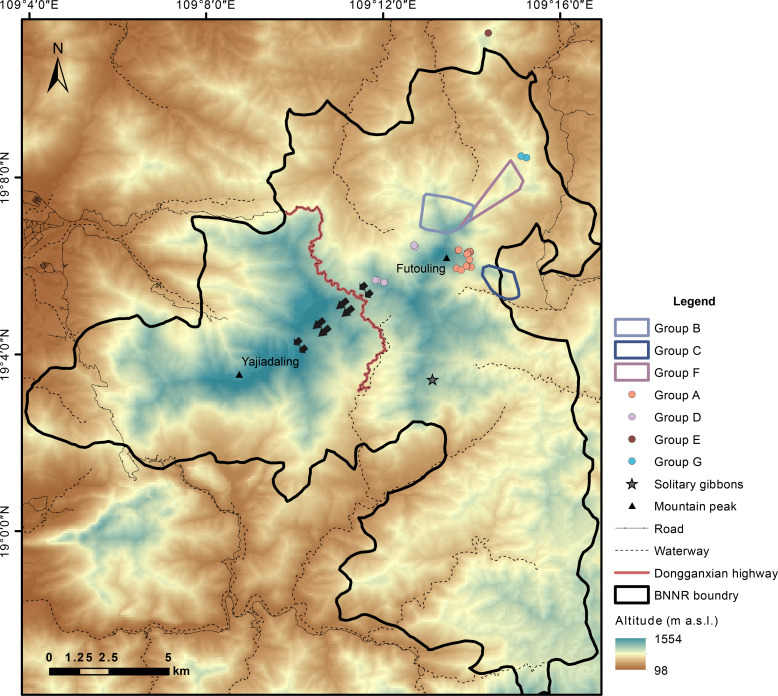
The distribution of the current Hainan gibbon population. The home range of groups B, C, and F were delineated using the 100% minimum convex polygon method based on location data collected at 30-min intervals during field tracking (home range size: GB-246.7 ha with 1260 location points, GC-132.5 ha with 1407 location points, and GF-186.7 ha with 59 location points). For the other gibbon groups, we did not collect sufficient location points to delineate their home range; thus, only a few observed points were presented. We displayed the boundary of the former Bawangling National Nature Reserve (BNNR; from 2003 to 2021) and the distribution of local roads and waterways (data sourced from OpenStreetMap). The highway, which hinders the dispersal of the Hainan gibbon within the nature reserve, is prominently highlighted and bold. The remaining Hainan gibbons are mainly found in higher-elevation areas (blue and beige pattern), whereas lower-elevation regions (brown pattern) are predominantly occupied by humans or have been transformed into large-scale plantations (*22*, *37*). “Yajiadaling” was once occupied by gibbons until the 1990s (*19*), and there are substantial expanses of primary forest remaining in this region (*37*). The arrows indicate our proposed construction of corridors to facilitate the dispersal of gibbons from “Futou Ling” to “Yajia Daling”. m a.s.l., meter above sea level.

Being a flagship species of the Hainan Tropical Rainforest National Park has made substantial funding available for habitat restoration for Hainan gibbons (*16*). Current restoration efforts have focused on enhancing the quality of existing habitats, while concurrently undertaking the restoration of low-land habitats (*57*). However, we have found that the existing habitats can effectively support the energy intake and reproductive capabilities of Hainan gibbons. This suggests that improving existing habitats would be a costly endeavor with limited effectiveness. Meanwhile, low-land habitats around the current Hainan gibbon population have been predominantly occupied by humans or transformed into large-scale pine and rubber plantations (*22*, *37*). Restoring extensive low-land habitats would be a time-consuming and costly process. Therefore, low-land habitat restoration is better considered a long-term strategy rather than an immediate solution to address the pressing need for expanding Hainan gibbon habitat. An alternative approach is available. Habitat fragmentation has confined the remaining population to an area of approximately 14 km^2^ (*37*, *57*). Plans to enhance habitat connectivity have been proposed for years but not effectively implemented. On the western side of the current habitat, there is a large forest patch that was once occupied by gibbons until the 1990s (*19*). This forest patch encompasses roughly 10 km^2^ of primary or old secondary forest (*37*), yet it is isolated from the current Hainan gibbon population by a highway (Fig. 4) (*19*, *57*). By constructing a corridor connecting existing habitat to this forest patch (Fig. 4), the available habitat could be swiftly expanded almost twofold. Hainan gibbon has been shown to use artificial canopy bridges (*58*), which could be used to construct these corridors. Strategies to attract gibbon dispersal through the corridor, such as playback calls, also need to be developed. Meanwhile, it should be noted that a great portion of the forest patch is located at a relatively high altitude, exceeding 1000 m above sea level, which may pose a notable risk of cold stress to Hainan gibbons (*59*). Therefore, it is imperative to evaluate in advance whether these high-altitude forests can provide sufficient food resources to meet the energy requirements of Hainan gibbons. Concurrently, long-term physiological and behavioral monitoring of gibbon groups that relocate to high-altitude habitats is essential to ensure their sustained survival in these environments.

### Captive breeding program for Hainan gibbons should be carefully considered

Natural recovery is exceedingly rare for extremely small animal populations. The presence of an extinction vortex can lead to population collapse, even when the average population growth rate is positive (*60*). Drawing from successful conservation efforts such as the crested ibis (*2*, *3*) and the black-footed ferret (*Mustela nigripes*) (*61*), captive breeding has been shown to be an effective approach for population recovery. Consequently, the incorporation of captive breeding into the Hainan gibbon rescue and protection action plan has been proposed for serious consideration.

Is captive breeding truly essential for the Hainan gibbon? Captive yellow-cheeked gibbons indeed showed a significantly shortened breeding intervals compared to their wild counterparts, suggesting the potential effectiveness of captive feeding for gibbons. However, the primary challenge of captive gibbon breeding lies in the low mating success among adults; thus, understanding the mechanism of mate choice for adult gibbons is a crucial prerequisite for successfully implementing captive breeding programs. Furthermore, capturing wild arboreal gibbons entails substantial risks of injury and deaths. Given the persistently low level of genetic diversity, the capture of wild individuals may further exacerbate the overall reduction in genetic diversity among wild Hainan gibbons, thereby exacerbating the inbreeding crisis. Therefore, while captive breeding can be considered a tool to mitigate extinction risk, we recommended first prioritizing the development of effective capture and mating techniques with other *Nomascus* gibbon species that have larger populations.

## MATERIALS AND METHODS

### Study site and subjects

The Hainan gibbon population contains seven family groups (GA, GB, GC, GD, GE, GF, and GG) and several solitary individuals, distributed in the Bawangling area (18°48′N to 19°12′N, 108°55′E to 109°17′E) of the HTRNP, Hainan Province, China. The area is a tropical evergreen rainforest with varying degrees of human disturbance. We placed a HOBO Automatic Rain Recorder (RG3-M) at the Shizigang monitoring station (altitude 904 m) to collect temperature and rainfall data throughout 2022. The annual average temperature was 19.6°C, while the annual precipitation was 1910 mm. The area has a distinct wet season from May to October, during which the average temperature was 21.8°C, and 83.7% of the total annual precipitation was recorded. The dry season is from November to April and has an average temperature of 17.7°C and an annual precipitation of merely 311 mm.

Behavioral data collection focused on group GB and GC, which were habituated [which were tracked to their sleeping trees (*62*)] through continuous tracking from March to December in 2021 (*32*). Group GB consisted of six to seven individuals including one to two adult male, two adult females, one to zero subadult, two juveniles, and one infant, while group GC consisted of seven to eight individuals including two adult males, two adult females, one to zero subadult, and three juveniles. Reproductive events were observed in adult females from both gibbon groups during the study. One female of group GB breed from January to May in 2022, while another was pregnant from September to December in 2022. One female of group GC was pregnant from June to November and breeding in December in 2022, while another adult female did not produce offspring in 2022.

### Behavioral data collection

We used a 5-min interval scan sampling (*63*) to collect behavioral data on calling, feeding, resting, traveling, and social behavior, as defined by Fan *et al.* (*64*). From December 2021 to December 2022, we monitored each group for approximately 50 hours per month, resulting in a behavioral dataset comprising 59,771 records (including 17,249 feeding records) during 1344 hours over 179 days. When gibbons were feeding, we recorded the food species and part eaten, including fruits, figs, leaves, buds, flowers, stems, seeds, and animal food (e.g., invertebrates and bird eggs). Concurrently, we used a 1-min focal animal sampling to estimate gibbon’s feeding rate for a specific food item. The focal sampling interval for the same individuals lasted at least 5-min. We collected 2224 feeding rate records on 190 food items from 102 plant species. This accounted for 90% of their annual feeding. We also recorded the group’s location with a hand-held GPS device (Garmin GPSMAP 639csx) at 5-min intervals. A full-day follow is defined as tracking the gibbon group from their sleeping tree (site) the day before to their sleeping tree (site) at the end of the day (*65*). We obtained 75 full-day follow (four to nine full days per month) and estimated the daily activity duration and the daily path length (DPL) [using the “move” package in R V.4.4.1 (*66*)].

### Food sample collection and nutritional analysis

We collected food plants consumed by the gibbons each month. Most of the plant samples were collected from the individual plants that our subjects had been observed to eat. A total of 137 food plant samples from 105 plant species, accounting for 87% of the annual feeding proportion, were collected for nutritional analysis. For each sample, we selectively picked out the parts that were eaten and measured the average fresh weight per unit (5 to 10 replicates). Then, we dried the samples in a blast drying oven at 45°C until reaching a constant weight (with a difference of less than 0.01 g between the two weighing measurements). We stored the dry samples in a sealed bag with silicone desiccant and sent for nutritional analysis. In the laboratory, we used an “intermittent grinding” method to grind all samples in a mill until they passed through 1-mm sieves. Sample particles with diameters between 0.25 and 1 mm were retained for nutritional analysis.

We analyzed the macronutrient composition of each food sample, encompassing available protein (AP), crude fat (CF), neutral detergent fiber (NDF), acid detergent fiber (ADF), crude ash (Ash), and total nonstructural carbohydrate (TNC) content. The CF (%) was determined by conducting petroleum ether extraction (using a SOX-416, Gerhardt) and subtracted one from it to approximately exclude nonfat components that are extracted by ether (*67*). If the ether extract of a sample was below 1%, then it was converted to 0. The NDF (%) and ADF (%) were sequentially determined following standardized detergent fiber analysis protocols (using a FT-12, Gerhardt) (*68*). The nitrogen content was determined using the standard Kjeldahl method (using a Vapodest-50S, Gerhardt). The total nitrogen content in a sample was determined and multiplied by 6.25 to estimate crude protein (CP) content. The nitrogen content in the sample that had completed the steps for measuring ADF was determined and multiplied by the same conversion factor to estimate the indigestible protein content (*69*, *70*). The AP (%) in a sample was estimated by subtracting the indigestible protein content from the CP (%). The food samples were burned in a muffle furnace at 250°C for 2 hours (with the furnace door open) and at 550°C for an additional 4 hours (with the furnace door closed) to determine Ash (%). The TNC (%) was calculated by subtracting CF (%), CP (%), NDF (%), and Ash (%) from 100% (*70*, *71*). All nutritional analysis experiments were conducted using 45°C dry samples with two replicates. If the relative SD of the two replicates exceeded 5%, then an additional replicate would be added. We further subjected 45°C dry samples to oven drying at 105°C for 16 hours to determine the dry matter mass per unit. The experimental values were transformed into mass percentages of 105°C dry matter, representing the macronutrient content in a sample.

### DEI estimate

Based on the behavioral data, we calculated the proportion of time that a specific sex class in a gibbon group spent on an activity based on the number of behavioral records for the activity divided by the total number of behavioral records. We calculated the feeding proportion for a specific sex class in a gibbon group toward a food item within a month by the number of feeding records for the food item divided by the total number of feeding records. The number of total behavioral records and feeding records for a specific sex class of a gibbon group within a month exceeded 250 and 60, respectively. The daily *DMI* (g·day^−1^) of a specific sex class in a gibbon group towards food item *i* was estimated as follows

$${\text{DMI}}_{i}=T_{\text{feed}}*60\text{DAD}*{\text{FP}}_{i}*{\text{DM}}_{i}*{\text{FR}}_{i}$$

where *T*_feed_ was the time proportion spent on feeding, *DAD* was the average daily activity duration (hour·day^−1^), *FP_i_* was the feeding proportion toward food item *i*, *DM_i_* was the dry mater mass per unit of food item *i* (g·unit^−1^), and *FR_i_* was the feeding rate of gibbons toward food item *i* (unit·min^−1^).

We estimated the metabolizable energy of each food item by summing the protein, fat and carbohydrate energy content with conversion factors of 4 kcal·day^−1^ for AP, 9 kcal·day^−1^ for CF, 4 kcal·day^−1^ for TNC, and 3 kcal·day^−1^ for NDF (*70*). Gibbons exhibit a limited capacity for fiber digestion and only partially convert dietary fiber into metabolic energy (*72*). We used a low-fermentation digestibility coefficient of 0.181, as used by Hon *et al.* (*73*), to estimate the dietary fiber energy intake of gibbons. The *DEI* (kcal·day^−1^) of a specific sex class in a gibbon group within a month was estimated as follows

$$\text{DEI}=\sum_{i=1}^{n}{\text{DMI}}_{i}*\left(4*{\text{AP}}_{i}+9*{\text{CF}}_{i}+4*{\text{TNC}}_{i}+3*0.181*{\text{NDF}}_{i}\right)$$

where *i* was food item consumed by Hainan gibbon. We calculated the average nutritional content values, categorized by plant parts, to estimate the macronutrient composition of food items belonging to the corresponding plant parts but lacking nutritional analysis. We conducted nutritional analysis of mealworms, which are commonly fed to gibbons in zoos, to estimate the macronutrient composition of animal food consumed by Hainan gibbon.

We used a single-sample t-test to estimate 95% CIs for daily DMI, daily macronutrient intake, and DEI of the Hainan gibbon throughout 2022 (*N* = 48). We further used a multivariable linear regression model to test for significant differences in DEI between different groups, sexes, or seasons, with DEI as dependent variable and group, sex, and season as independent variables. The dependent variable was logarithmically transformed to ensure that the residuals of the model conform to a normal distribution.

### DEE estimate

The *BMR* (kcal·day^−1^) serves as the fundamental basis for estimating the animal’s DEE across various activities and is highly correlated with *BW* (kg), *BMR* = 70*BW^0.75^ (*71*, *74*). BW data are difficult to measure for wild arboreal gibbons. Therefore, we used the mean *BW* of Hainan gibbons as reported by Liu (*38*) and Xu (1983) (*39*), who obtained six specimens (three females and three males) in the wild, to estimate BW. The estimated BWs of adult female and male Hainan gibbons were 6.56 and 7.74 kg, respectively. Adolescent individuals were excluded from the estimation of the DEE due to fluctuations in their BWs during growth and development.

The DEE for locomotion (*E*_loc_, kcal·day^−1^) in gibbons was estimated on the basis of *BW*, *DPL* (km·day^−1^), the proportion of time spent on locomotion activities (*T*_loc_), and daily activity duration (*75*)

$$\begin{array}{c} E_{\text{loc}}=0.041*{\left(10^{3}\text{BW}\right)}^{0.60}*\text{DPL}\\ +0.029*{\left(10^{3}\text{BW}\right)}^{0.75}*T_{\text{loc}}*\text{DAD} \end{array}$$

The *DEE* (kcal·day^−1^) of gibbons was estimated by the cumulative sum of the energy expenditure for sleeping and various daily activities (*76*)

$$\text{DEE}=\left[\text{BMR}*\left(1-\frac{\text{DAD}}{24}\right)\right]+\sum_{i}^{n}\left(D_{i}*\text{BMR}*T_{i}*\frac{\text{DAD}}{24}\right)+E_{\text{loc}}$$

where *D_i_* was the energy constant for different activity, with *D*_rest_ = 1.25, *D*_feed_ = 1.38, and *D*_social_ = 2.35 (*77*). The calling behavior of gibbons is energetically demanding and serves an important social purpose (*78*); thus, we used the same energy constant as social behavior. Adult females have additional energy expenditure during reproductive events. Female primates experience an average increase of 25% in their DEE during pregnancy and approximately 50% during lactation (*79*). Therefore, we estimated that the DEE of female Hainan gibbons was 1.25 times higher than the values calculated using the above formula during pregnancy and 1.5 times higher during lactation.

We further calculated daily *NEI* (kcal·d^−1^) by subtracting DEE from DEI to assess the potential presence of an energy budget bottleneck in the two groups during different seasons. We used a single-sample *t* test to estimate 95% CIs for DEE and NEI of the Hainan gibbon throughout 2022 (*N* = 48) and for NEI of a Hainan gibbon group in a season (*N* = 12). We used a multivariable linear regression model to test for significant differences in NEI between different groups, sexes, or seasons, with NEI as dependent variable and group, sex, and season as independent variables. The dependent variable was logarithmically transformed to ensure that the residuals of the model conformed to a normal distribution.

### Estimation of daily macronutrient and metabolic energy for captive gibbons

We acquired the daily feeding diet for captive yellow-cheeked gibbons (*N. gabriellae*) from four zoos in China, namely, the Guangzhou, Kunming, Nanjing, and Nanning Zoos. A total of 60 food samples (dried in a blast drying oven at 45°C), which were fed to the gibbons, were provided by these zoos. Subsequent to grinding, we conducted nutritional analysis consistent with those performed on wild gibbon food samples. On the basis of the quantified feeding diet provided by the zoos and the nutritional composition of the food samples, we estimated the daily macronutrients and metabolic energy supplied to captive yellow-cheeked gibbons by each zoo.

### IBI and MDA assessments

We assessed IBI and MDA of Hainan gibbons based on population data reported by Zhou *et al.* (*28*) and Deng *et al.* (*80*) covering the period from 2003 to 2013, as well as the long-term field records of the Bawangling Branch of Hainan Tropical Rainforest National Park Administration from 2013 to 2024 (tables S4 and S5). The IBI was determined as the duration between two successive breeding events. Gibbons typically lactate for 1.5 years, and IBI shorter than 1.5 years may arise from infanticide or premature mortality, which are not taken into account in the calculation of IBI. The MDA was determined by the length of time from birth to the gibbon’s dispersal from its natal group. We applied the same assessment criteria to estimate IBI in wild populations of Cao vit gibbon (*N. nasutus*), and western black-crested gibbon (*N. concolor*) based on our long-term continuous monitoring of populations from 2003 to 2024 for *N. concolor* in “Dazaizi” at Mt. Wuliang, Yunnan, China and from 2008 to 2024 for *Nomascus nasutus* in Bangliang, Guangxi, China (table S4). We also calculated the IBI of the captive yellow-cheeked gibbons in the four zoos based on the reproductive data reported by Fan *et al.* (*81*) covering the period from 2003 to 2018. The four captive gibbon populations are in good health and reproduce well (*81*). The LMM to test for significant differences in the IBI between the Hainan gibbons and the other three gibbon species was implemented using the R package “lme4” (*82*). Besides, we collected the average IBI and MDA of 10 gibbon species from literature sources (table S2). In cases where multiple literature sources were available, we performed a weighted average calculation based on the number of reproductive events.

### Fecal sample collection and DNA extraction

Between March 2021 and December 2022, and in April 2024, we collected 106 fresh fecal samples from five Hainan gibbon groups and a solitary female (GA: *N* = 2, GB: *N* = 41, GC: *N* = 40, GE: *N* = 10, GF: *N* = 9, solitary female: *N* = 4). Immediately after collection, all fecal samples were stored in 99.9% ethanol, and each sample was subsequently desiccated using silica after more than 24 hours of storage in 99.9% ethanol (*83*). All samples were then stored at −20°C in the laboratory for long-term preservation. The total genomic DNA of each sample was extracted using a QIAamp Fast DNA Stool Mini kit (QIAGEN, Germany), following the manufacturer’s protocol with an additional peri-extraction enrichment step using SDS (*84*). The quality of the extracted DNA was quantified using a NanoDrop ND-1000 spectrometer (Thermo Fisher Scientific).

### Microsatellite loci genotyping

We genotyped 10 microsatellite loci used in Guo *et al.* (*35*): SSR12, SSR17, D2S367, D5S1457, D7S817, D1S548, D5S1470, D6S265, D20S206, and DQcar. The polymerase chain reaction (PCR) amplifications were performed in a reaction volume of 10 μl, containing 2× Quick Taq HS DyeMix (TOYOBO, Japan), 2.5 mM MgCl_2_, 0.25 μM of each primer with forward primers fluorescently labeled (three to four pairs of primers mixed), 1 μl of bovine serum albumin (BSA) (10 mg/ml), and 2 μl of template DNA. We used touchdown PCR to enhance the specificity of primer annealing and DNA production. The PCR cycling conditions comprised an initial denaturation step at 94°C for 15 min, followed by 20 cycles of denaturation at 94°C for 30 s, annealing at 58°C for 20 s (decreasing by 0.5°C per cycle), and extension at 72°C for 30 s; then 20 cycles of denaturation at 94°C for 30 s, annealing at 48°C for 30 s, and extension at 72°C for 30 s; and lastly, a final extension step at 72°C for 10 min. To prevent contamination, a negative control was processed alongside each set of PCRs. We confirmed genotypes from more than two duplicate samples and employed a multiple-tube replication procedure to minimize the likelihood of allelic dropout and genotyping errors when amplifying microsatellite loci using fecal samples (*85*, *86*). The PCR products were electrophoresed on an ABI 3730xl genetic analyzer (Applied Biosystems) at Sangon Biotech Company in Shanghai, China.

### Sex determination

We used two loci, AMEL and SRY, to determine the sex of the Hainan gibbon. These loci had previously been used for sex determination from fecal samples of *Nomascus* gibbons (*87*, *88*). PCR amplifications were performed in a 10-μl reaction volume, containing 2× Quick Taq HS DyeMix (TOYOBO, Japan), 2.5 mM MgCl_2_, 0.4 μM of each primer with forward primers fluorescently labeled (two pairs of primers mixed), 1 μl of BSA (10 mg/ml), and 2 μl of template DNA. The PCR conditions were as follows: an initial denaturing at 94°C for 15 min, then 35 cycles of denaturation at 94°C for 30 s, followed by annealing at 58°C for 45 s, extension at 72°C for 45 s, and a final extension at 60°C for 30 min. All PCR products were sent to Sangon Biotech Company in Shanghai, China and sequenced on an ABI 3730xl genetic analyzer.

### D-loop sequencing

We used GDL-L1 and GDL-H2 to amplify the mitochondrial DNA (mtDNA) D-loop gene from different individuals (*89*). PCR amplifications were performed in a 20 μl reaction volume containing 1 to 2 μl of template DNA (50 to 100 ng), 2 μl of 10× buffer,1.6 μl of deoxynucleoside triphosphate (2.5 mM each), 1 μl of each primer (10 μM), 0.2 μl BSA (10 mg/ml), and 0.1 μl of TaKaRa Ex Taq (5 U/μl). The PCR conditions comprised an initial denaturing at 91°C for 2 min, followed by 35 cycles of denaturation at 95°C for 15 s, annealing at 58°C for 30 s, and extension at 70°C for 15 s, with a final extension step at 60°C for 10 min. All PCR products were sent to Sangon Biotech Company in Shanghai, China and sequenced on an ABI 3730xl genetic analyzer.

### Genetic analyses

The genotyping analysis for microsatellite loci and sexual loci was performed against an internal size standard GeneScan 500 LIZ (Applied Biosystems) using GeneMarker v. 1.91. Individuals of group A and group D were extracted from the other two studies that were based on same microsatellite loci (*35*, *42*). We conducted identity analysis in CERVUS version 3.0.7 (*90*). Scoring errors in the merged genotype data were tested using MICRO-CHECKER v. 2.2.3 (*91*). GenAlEx 6.502 (*92*) was used to calculate the number of different alleles (Na) across the 10 loci. Observed heterozygosity (*H*_O_), expected heterozygosity (*H*_E_), inbreeding coefficient (*F*_IS_), Hardy-Weinberg equilibrium, and pairwise linkage disequilibrium were tested using ARLEQUIN version 3.5 (*93*). The *P* value of Hardy-Weinberg equilibrium and pairwise linkage disequilibrium were adjusted using the Bonferroni test (*94*). One pair of loci exhibited pairwise linkage disequilibrium, indicating that inheritance by descent was not independent. This could potentially affect the calculation of the relatedness coefficient. Therefore, we excluded one locus, D6S265, which showed linkage disequilibrium to other four loci before Bonferroni test. This locus was excluded from further pairwise relatedness analyses. After excluding this locus, the remaining loci did not show pairwise linkage disequilibrium.

All mtDNA sequences were assembled using the DNAstar Lasergene Seqman Pro version 7.1.0. We downloaded a D-loop sequence of AM from GenBank: MW052605. Subsequently, the assembled sequences were aligned and compared using MEGA 7 (*95*). We calculated the number of haplotypes (h), haplotype diversity (Hd) and nucleotide diversity (Pi) of 24 individuals using Dnasp version 6.12.03 (*96*).

To analyze whether the genetic diversity was still decreasing over time, we compared the Na and *H*_E_ among individuals from different periods based on the same seven microsatellite loci. This comparison was conducted between our study and the study by Bryant *et al.* (*34*). For this purpose, we used the nonparametric paired Wilcoxon signed-rank test to test the significance of difference.

To understand the relatedness among breeding pairs (dyads) from different groups, we estimated the degree of relatedness using Wang’s unbiased estimator, which was particularly suitable for analyzing small sample sizes that may have related dyads (*97*, *98*). We used the software COANCESTRY version 1.0.1.8 (*99*) to perform these calculations. Within our analysis, we treated each male-female dyad’s pairwise relatedness as an independent case. We assigned integer values of 1 to the original Hainan gibbon groups (GA and GB), while integer values ranging from two to five were assigned to the group GC to GF based on their formation time from font to back. The pairwise relatedness between intermating pairs was set as dependent variable, while the integer values reflecting the temporal sequence of group formation was set as independent variable. Then, we used a linear regression model to test for the correlation between these two variables (*N* = 14).

### Statistical analyses

Statistical analyses were all performed in R (version 4.4.1). The details have been explained in Results and Materials and Methods.

## Supplementary Materials

### This PDF file includes:

Fig. S1 Tables S1 to S5 References
