# Supplementary material for: Science-based suggestions to save the world’s rarest primate species Nomascus hainanus
Source: Sci Adv. 2025 Apr 11;11(15):eadv4828. doi: 10.1126/sciadv.adv4828 (PMC11988426; doi:10.1126/sciadv.adv4828)
Supplement: Supplementary file 1 — Fig. S1 Tables S1 to S5 References [file sciadv.adv4828_sm.pdf]

Science Advances
AAAS

Supplementary Materials for

Science-based suggestions to save the world's rarest primate species *Nomascus hainanus*

Xukai Zhong *et al.*

Corresponding author: Pengfei Fan, fanpf@mail.sysu.edu.cn

Sci. Adv. **11**, eadv4828 (2025)
DOI: 10.1126/sciadv.adv4828

This PDF file includes:

Fig. S1
Tables S1 to S5
References

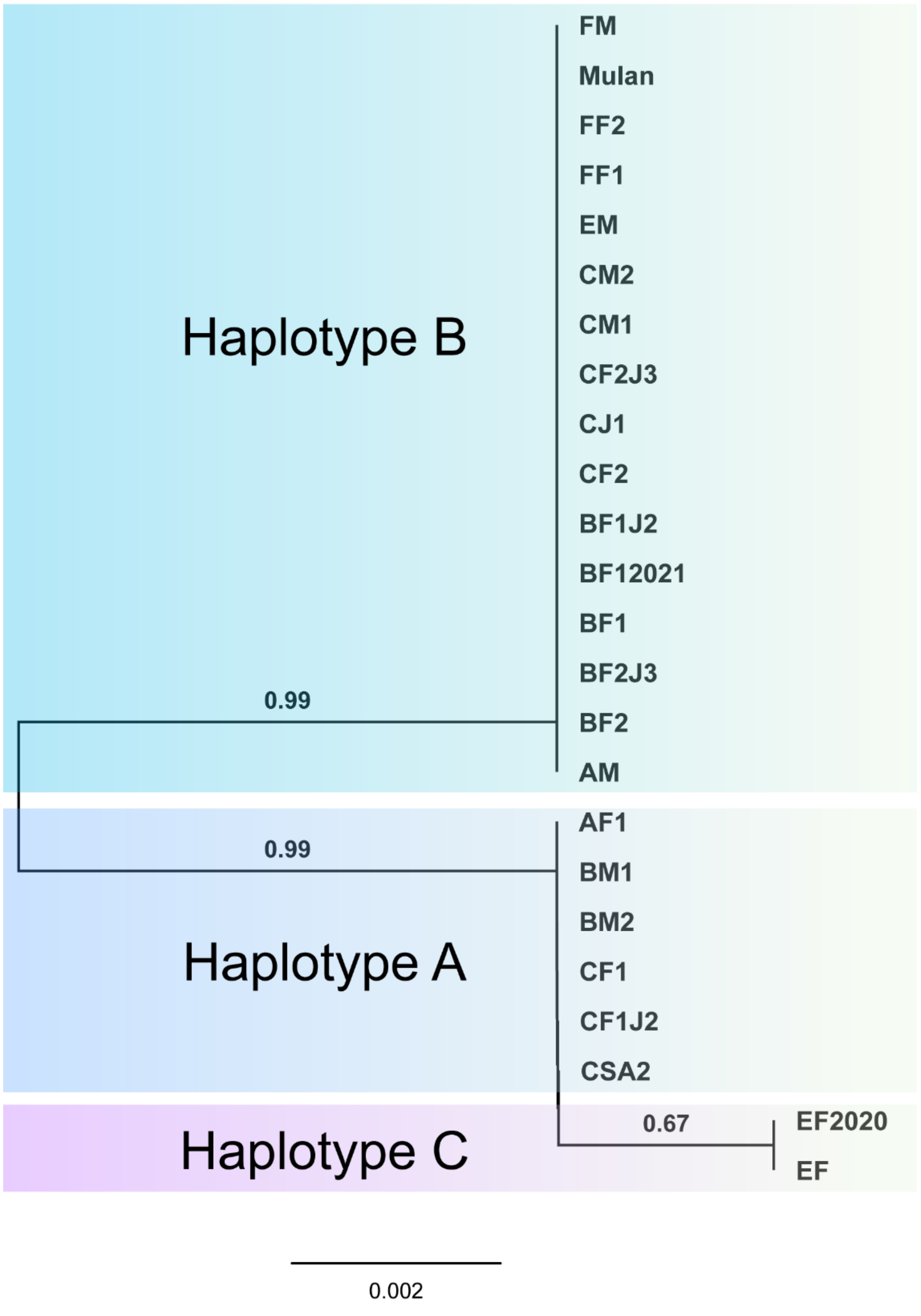

Figure S1. The neighbor-joining tree based on the d-loop sequences of 24 Hainan gibbon individuals.

Table S1. The ratio of daily energy intake to basal metabolic rate for 11 categories of captive free-feeding primates. The data on the body weight (*BW*, kg), daily energy intake (*DEI*, kcal·d^{-1}), and basal metabolic rate (*BMR*, kcal·d^{-1}) of these primates originate from the National Research Council (2003) (*40*). Due to the inability to accurately assign some captive primates to specific species, we present only primate categories along with their corresponding genus names. For primates with more than one dataset, we present values weighted by the number of individuals in each dataset.

Primate	Genus	*BW* (kg)	*DEI* (kcal·d^{-1})	*BMR* (kcal·d^{-1})	*Ratio*
Great Ape	*Gorilla*, *Pongo*	76.50	2,583.00	1,810.69	1.43
Baboon	*Papio*	17.80	926.27	606.61	1.53
Rhesus monkey	*Macaca*	10.72	588.04	414.82	1.42
Proboscis Monkey	*Nasalis*	10.00	1,172.17	393.64	2.97
Howler Monkey	*Alouatta*	7.57	435.00	319.30	1.36
Long-tailed Macaque	*Macaca*	5.60	529.5	254.82	2.08
Lemur	*Lemur*	3.11	222.39	163.76	1.35
Aye-aye	*Daubentonia*	2.46	260.00	137.00	1.90
Squirrel Monkey	*Saimiri*	0.83	164.80	61.02	2.70
Tamarin	*Saguinus*	0.49	100.84	40.73	2.48
Marmoset	*Callithrix*, *Saguinus*	0.33	66.35	30.79	2.15

Table S2. Female interbirth intervals and male dispersal ages of 10 gibbon species. We present the mean, standard deviation (*SD*), and sample size (*N*) for both Interbirth interval (*IBI*, years) and male dispersal age (*MDA*, years) for each gibbon species. "-" indicates that the data is missing from the source literature.

Species	*Mean IBI*	*SD*1	*N*1	*Mean MDA*	*SD*2	*N*2	Source
Hoolock hoolock	3	-	-	6	-	-	(*100*)
Hylobates klossii	3.30	2.30	6	8	-	-	(*100, 101*)
Hylobates albibarbis	3.20	0.40	5	8.8	-	-	(*102, 103*)
Hylobates agilis	3.83	1.15	4	8	-	-	(*103*)
Hylobates lar	3.42	0.76	17	9.74	0.68	6	(*104, 105*)
Hylobates moloch	3.58	-	-	9	-	-	(*106, 107*)
Symphalangus syndactylus	2.78	0.25	7	8.5	-	-	(*100, 108, 109*)
Nomascus concolor	3.32	0.77	24	9.91	1.45	11	This Study
Nomascus nasutus	3.20	0.84	20	9.5	0.58	4	This Study
Nomascus hainanus	2.80	0.98	25	6.90	1.14	5	This Study

Table S3. Microsatellite genotypes and sexes of 31 individuals of Hainan gibbons.

Individual ID	Description	Group	Sex	D2S367		D5S1457		NL-SSR12		D1S548		D20S206		Source
AF1	Adult breeding female	GA	Female	144	144	108	112	182	188	163	167	132	132	This study
AM	Adult breeding male	GA	Male	144	154	108	108	182	182	167	175	132	144	(*35*)
AM3	Adult male	GA	Male	144	154	108	112	182	182	167	175	132	144	(*42*)
AF2	Adult breeding female	GA	Female	144	144	108	112	182	188	163	163	132	132	(*42*)
BF2	Adult breeding female	GB	Female	144	154	108	108	182	188	167	175	132	132	This study
BF1	Adult breeding female	GB	Female	144	154	108	108	182	182	167	175	132	144	This study
BF2J3	Offspring	GB	Male	144	154	108	108	182	188	167	175	132	132	This study
BF12021	Offspring	GB	Female	144	154	108	108	182	182	167	175	132	132	This study
BF1J2	Offspring	GB	Male	144	154	108	108	182	182	175	175	132	144	This study
BM1	Adult breeding male	GB	Male	144	144	108	108	182	188	167	167	132	132	This study
BM2	Adult breeding male	GB	Male	144	154	108	112	188	188	163	163	132	132	This study
CF1	Adult breeding female	GC	Female	144	144	108	112	182	188	163	167	132	132	This study
CF1J2	Offspring	GC	Male	144	144	108	112	188	188	167	175	132	132	This study
CF2	Adult breeding female	GC	Female	144	154	108	108	182	182	167	175	132	132	This study
CJ1	Offspring	GC	Female	144	154	108	108	182	188	163	167	132	132	This study
CF2J3	Offspring	GC	Male	144	144	108	108	182	188	175	175	132	132	This study
CM1	Adult breeding male	GC	Male	144	144	108	108	182	188	175	175	132	132	This study
CM2	Adult breeding male	GC	Male	144	154	108	108	188	188	163	175	132	144	This study
DJ1	Offspring	GD	Male	144	154	108	108	182	188	167	175	132	144	(*42*)
DM1	Adult breeding male	GD	Male	144	154	108	108	182	188	167	175	132	144	(*42*)
DM2	Adult breeding male	GD	Male	144	154	108	108	182	188	163	167	132	144	(*42*)
DF1	Adult breeding female	GD	Female	144	148	108	108	182	188	163	163	132	144	(*42*)
EM	Adult breeding male	GE	Male	144	154	108	108	182	182	167	167	132	132	This study
EF	Adult breeding female	GE	Female	144	144	108	108	182	188	163	167	132	132	This study
EF2020	Offspring	GE	Male	144	154	108	108	182	182	167	167	132	132	This study
FM	Adult breeding male	GF	Male	144	154	108	108	182	188	167	175	132	132	This study
FF1	Adult breeding female	GF	Female	144	154	108	108	182	188	163	167	132	132	This study
FF2	Adult breeding female	GF	Female	144	154	108	108	182	182	167	175	132	132	This study
Mulan	Solitary adult female	GL	Female	144	154	108	108	182	188	167	175	132	144	This study
CSA2	Solitary sub-adult male	GL	Male	144	154	108	108	182	188	163	167	132	132	This study
CSA1	Solitary sub-adult male	GL	Male	144	144	108	112	188	188	163	175	132	132	(*35*)

Individual ID	Description	Group	Sex	DQcar		D5S1470		D7S817		NL-SSR17		D6S265		Source
AF1	Adult breeding female	GA	Female	106	106	197	201	179	183	216	220	128	136	This study
AM	Adult breeding male	GA	Male	92	106	197	197	179	183	212	220	120	130	(*35*)
AM3	Adult male	GA	Male	92	106	197	197	179	183	212	220	120	130	(*42*)
AF2	Adult breeding female	GA	Female	92	106	197	197	179	183	212	216	120	128	(*42*)
BF2	Adult breeding female	GB	Female	92	106	197	197	179	179	212	216	120	128	This study
BF1	Adult breeding female	GB	Female	92	106	197	197	179	179	216	220	128	130	This study
BF2J3	Offspring	GB	Male	106	106	197	201	179	183	212	220	120	128	This study
BF12021	Offspring	GB	Female	92	106	197	197	179	179	212	220	128	136	This study
BF1J2	Offspring	GB	Male	92	106	197	201	179	179	212	220	120	128	This study
BM1	Adult breeding male	GB	Male	92	106	197	201	179	183	212	220	120	136	This study
BM2	Adult breeding male	GB	Male	92	106	197	197	179	183	212	220	128	130	This study
CF1	Adult breeding female	GC	Female	92	106	197	197	179	183	212	220	128	130	This study
CF1J2	Offspring	GC	Male	92	106	197	197	179	183	212	212	128	130	This study
CF2	Adult breeding female	GC	Female	106	106	197	197	179	183	220	220	128	136	This study
CJ1	Offspring	GC	Female	92	106	197	197	179	183	212	220	130	136	This study
CF2J3	Offspring	GC	Male	106	106	197	197	179	183	212	220	130	136	This study
CM1	Adult breeding male	GC	Male	92	106	197	197	179	183	212	220	130	136	This study
CM2	Adult breeding male	GC	Male	92	106	197	197	179	179	212	220	130	130	This study
DJ1	Offspring	GD	Male	0	0	197	201	179	179	216	220	120	130	(*42*)

DM1	Adult breeding male	GD	Male	0	0	197	201	179	179	220	220	120	130	(*42*)
DM2	Adult breeding male	GD	Male	0	0	197	201	179	183	216	220	0	0	(*42*)
DF1	Adult breeding female	GD	Female	0	0	197	197	179	179	220	220	130	130	(*42*)
EM	Adult breeding male	GE	Male	92	106	197	201	179	179	220	220	120	130	This study
EF	Adult breeding female	GE	Female	92	106	197	197	179	179	212	220	130	130	This study
EF2020	Offspring	GE	Male	92	106	197	201	179	179	212	220	120	130	This study
FM	Adult breeding male	GF	Male	92	106	197	201	179	183	212	220	120	128	This study
FF1	Adult breeding female	GF	Female	92	106	197	197	179	183	220	220	130	136	This study
FF2	Adult breeding female	GF	Female	92	106	197	201	179	183	220	220	120	130	This study
Mulan	Solitary adult female	GL	Female	92	106	197	201	179	183	212	216	120	130	This study
CSA2	Solitary sub-adult male	GL	Male	92	106	197	201	179	183	220	220	130	136	This study
CSA1	Solitary sub-adult male	GL	Male	92	106	197	197	179	183	212	220	128	130	(*35*)

Table S4. Interbirth events of female individuals among four *Nomascus* gibbon populations. Each line of data represents an interbirth event. The data of wild Hainan gibbon (*N. hainanus*), Cao vit gibbon (*N. nasutus*), western black-crested gibbon (*N. concolor*), and captive yellow-cheeked gibbon (*N. gabriellae*) span from 2003 to 2024, from 2008 to 2024, from 2003 to 2024, and from 2003 to 2018, respectively.

Species	Population	Individual ID	*IBI* (years)	Source
Nomascus hainanus	"Bawangling"	AF1	1.92	(*80*)
Nomascus hainanus	"Bawangling"	AF1	3.00	This Study
Nomascus hainanus	"Bawangling"	AF2	2.08	(*80*)
Nomascus hainanus	"Bawangling"	AF2	1.92	(*80*)
Nomascus hainanus	"Bawangling"	AF2	1.67	(*80*)
Nomascus hainanus	"Bawangling"	AF2	2.25	This Study
Nomascus hainanus	"Bawangling"	AF2	3.75	This Study
Nomascus hainanus	"Bawangling"	AF2	3.50	This Study
Nomascus hainanus	"Bawangling"	BF1	4.08	This Study
Nomascus hainanus	"Bawangling"	BF2	2.00	(*80*)
Nomascus hainanus	"Bawangling"	BF2	2.17	(*80*)
Nomascus hainanus	"Bawangling"	BF2	2.08	(*80*)
Nomascus hainanus	"Bawangling"	BF2	2.75	(*80*)
Nomascus hainanus	"Bawangling"	BF2	1.58	(*80*)
Nomascus hainanus	"Bawangling"	BF2	5.50	This Study
Nomascus hainanus	"Bawangling"	BF2	2.08	This Study
Nomascus hainanus	"Bawangling"	CF1	2.75	(*80*)
Nomascus hainanus	"Bawangling"	CF1	2.58	This Study
Nomascus hainanus	"Bawangling"	CF1	4.00	This Study
Nomascus hainanus	"Bawangling"	CF2	3.75	This Study
Nomascus hainanus	"Bawangling"	CF2	2.67	This Study
Nomascus hainanus	"Bawangling"	CF2	4.33	This Study
Nomascus hainanus	"Bawangling"	DF1	2.17	This Study
Nomascus hainanus	"Bawangling"	DF2	2.50	This Study
Nomascus hainanus	"Bawangling"	EF	2.92	This Study
Nomascus nasutus	"Bangliang"	G1F1	3.08	This Study
Nomascus nasutus	"Bangliang"	G1F1	4.00	This Study
Nomascus nasutus	"Bangliang"	G1F2	3.00	This Study
Nomascus nasutus	"Bangliang"	G1F3	3.00	This Study
Nomascus nasutus	"Bangliang"	G1F3	2.17	This Study
Nomascus nasutus	"Bangliang"	G1F3	5.00	This Study
Nomascus nasutus	"Bangliang"	G2F1	1.92	This Study
Nomascus nasutus	"Bangliang"	G2F1	2.92	This Study
Nomascus nasutus	"Bangliang"	G2F1	4.08	This Study
Nomascus nasutus	"Bangliang"	G2F3	2.00	This Study
Nomascus nasutus	"Bangliang"	G4F1	3.17	This Study
Nomascus nasutus	"Bangliang"	G4F1	3.75	This Study
Nomascus nasutus	"Bangliang"	G4F1	3.08	This Study
Nomascus nasutus	"Bangliang"	G4F1	3.08	This Study
Nomascus nasutus	"Bangliang"	G4F1	2.00	This Study
Nomascus nasutus	"Bangliang"	G4F2	2.67	This Study
Nomascus nasutus	"Bangliang"	GMF1	3.00	This Study
Nomascus nasutus	"Bangliang"	GMF2	4.25	This Study
Nomascus nasutus	"Bangliang"	GLF1	3.83	This Study

Nomascus nasutus	"Bangliang"	GLF2	4.08	This Study
Nomascus concolor	"Dazaizi"	G2AFR	3.08	This Study
Nomascus concolor	"Dazaizi"	G2AFR	2.67	This Study
Nomascus concolor	"Dazaizi"	G2AFR	2.75	This Study
Nomascus concolor	"Dazaizi"	G2AFR	4.58	This Study
Nomascus concolor	"Dazaizi"	G2AFR	3.33	This Study
Nomascus concolor	"Dazaizi"	G2FR2006	3.50	This Study
Nomascus concolor	"Dazaizi"	G2FR2006	2.50	This Study
Nomascus concolor	"Dazaizi"	G2FR2006	2.83	This Study
Nomascus concolor	"Dazaizi"	G3FB	3.25	This Study
Nomascus concolor	"Dazaizi"	G3FB	5.00	This Study
Nomascus concolor	"Dazaizi"	G3FY	4.42	This Study
Nomascus concolor	"Dazaizi"	G3FY	4.00	This Study
Nomascus concolor	"Dazaizi"	G3FY	2.58	This Study
Nomascus concolor	"Dazaizi"	G3FY	2.92	This Study
Nomascus concolor	"Dazaizi"	G3FY	2.25	This Study
Nomascus concolor	"Dazaizi"	G3FY	2.75	This Study
Nomascus concolor	"Dazaizi"	G4FB	3.83	This Study
Nomascus concolor	"Dazaizi"	G4FB	3.33	This Study
Nomascus concolor	"Dazaizi"	G4FB	2.58	This Study
Nomascus concolor	"Dazaizi"	G4FB	3.00	This Study
Nomascus concolor	"Dazaizi"	G4FB	3.00	This Study
Nomascus concolor	"Dazaizi"	G4FY	4.42	This Study
Nomascus concolor	"Dazaizi"	G4FY	2.75	This Study
Nomascus concolor	"Dazaizi"	G4FY	4.42	This Study
Nomascus gabriellae	Captive	NJF1	2.08	(*81*)
Nomascus gabriellae	Captive	NJF1	1.83	(*81*)
Nomascus gabriellae	Captive	NNF1	3.00	(*81*)
Nomascus gabriellae	Captive	NNF1	1.83	(*81*)
Nomascus gabriellae	Captive	NNF1	1.50	(*81*)
Nomascus gabriellae	Captive	NNF1	1.58	(*81*)
Nomascus gabriellae	Captive	NNF1	2.08	(*81*)
Nomascus gabriellae	Captive	NNF1	2.33	(*81*)
Nomascus gabriellae	Captive	NNF2	1.67	(*81*)
Nomascus gabriellae	Captive	NNF2	2.50	(*81*)
Nomascus gabriellae	Captive	NNF3	2.83	(*81*)
Nomascus gabriellae	Captive	NNF4	2.75	(*81*)
Nomascus gabriellae	Captive	NNF4	2.00	(*81*)

Table S5. Dispersal events of male individuals among three *Nomascus* gibbon populations. Each line of data represents a dispersal event. The data of wild Hainan gibbon (*N. hainanus*), Cao vit gibbon (*N. nasutus*), western black-crested gibbon (*N. concolor*) span from 2003 to 2024, from 2008 to 2024, and from 2003 to 2018, respectively.

Species	Population	Individual ID	Dispersal Age (years)	Source
Nomascus hainanus	"Bawangling"	Unknown	5.5	(*28*)
Nomascus hainanus	"Bawangling"	ASA	8	This Study
Nomascus hainanus	"Bawangling"	BSA	7	This Study
Nomascus hainanus	"Bawangling"	BJ1	6	This Study
Nomascus hainanus	"Bawangling"	CSA	8	This Study
Nomascus nasutus	"Bangliang"	G1F1-02	10	This Study
Nomascus nasutus	"Bangliang"	G4-02	10	This Study
Nomascus nasutus	"Bangliang"	G4-05	9	This Study
Nomascus nasutus	"Bangliang"	G4F2-10	9	This Study
Nomascus concolor	"Dazaizi"	SM2-05	11	(*41*)
Nomascus concolor	"Dazaizi"	SM2-06	8	(*41*)
Nomascus concolor	"Dazaizi"	SM2-14	11	(*41*)
Nomascus concolor	"Dazaizi"	G3FB1998	11	(*41*)
Nomascus concolor	"Dazaizi"	G3FB2001	11	(*41*)
Nomascus concolor	"Dazaizi"	SM3-05	10	(*41*)
Nomascus concolor	"Dazaizi"	G3FB2004	8	(*41*)
Nomascus concolor	"Dazaizi"	SM4-12	12	(*41*)
Nomascus concolor	"Dazaizi"	SM4-13	9	(*41*)
Nomascus concolor	"Dazaizi"	G4FB2006	8	(*41*)
Nomascus concolor	"Dazaizi"	G4FY2007	10	(*41*)

REFERENCES AND NOTES

1. G. Caughley, Directions in conservation biology. *J. Anim. Ecol.* **63**, 215–244 (1994).

2. Y. M. Xi, B. Z. Lu, Y. M. Zhang, N. Fujihara, Restoration of the crested ibis, *Nipponia nippon*. *J. Appl. Anim. Res.* **22**, 193–200 (2002).

3. F. Wang, M. Li, Y. S. Zhang, W. A. Zhao, D. N. Liu, Y. Z. Zhang, H. Zhang, X. P. Ye, X. P. Yu, Post-release dispersal and breeding site suitability of reintroduced populations of the Crested Ibis in Shaanxi Province, China. *Restor. Ecol.* **29**, e13383 (2021).

4. P. S. Alagona, Biography of a "Feathered Pig": The California condor conservation controversy. *J. Hist. Biol.* **37**, 557–583 (2004).

5. S. Turvey, *Witness To Extinction*. (Oxford Univ. Press, 2008).

6. R. Emslie. 2020. "*Ceratotherium simum*" (IUCN, 2020); https://dx.doi.org/10.2305/IUCN.UK.2020-1.RLTS.T4185A45813880.en.

7. W. F. Fagan, E. E. Holmes, Quantifying the extinction vortex. *Ecol. Lett.* **9**, 51–60 (2006).

8. R. Williams, R. C. Lacy, E. Ashe, L. Barrett-Lennard, T. M. Brown, J. K. Gaydos, F. Gulland, M. Macduffee, B. W. Nelson, K. A. Nielsen, H. Nollens, S. Raverty, S. Reiss, P. S. Ross, M. S. Collins, R. Stimmelmayr, P. Paquet, Warning sign of an accelerating decline in critically endangered killer whales (*Orcinus orca*). *Commun. Earth Environ.* **5**, 173 (2024).

9. J. M. Drake, B. D. Griffen, Early warning signals of extinction in deteriorating environments. *Nature* **467**, 456–459 (2010).

10. J. L. Godwin, A. J. Lumley, L. Michalczyk, O. Y. Martin, M. J. G. Gage, Mating patterns influence vulnerability to the extinction vortex. *Glob. Change Biol.* **26**, 4226–4239 (2020).

11. F. Palomares, J. Antonio Godoy, J. Vicente Lopez-Bao, A. Rodriguez, S. Roques, M. Casas-Marce, E. Revilla, M. Delibes, Possible extinction vortex for a population of iberian lynx on the verge of extirpation. *Conserv. Biol.* **26**, 689–697 (2012).

12. J. C. Z. Woinarski, A very preventable mammal extinction. *Nature* **535**, 493–493 (2016).

13. F. Palomares, A. Rodriguez, E. Revilla, J. Vicente Lopez-Bao, J. Calzada, Assessment of the conservation efforts to prevent extinction of the Iberian Lynx. *Conserv. Biol.* **25**, 4–8 (2011).

14. M. N. Clout, G. P. Elliott, B. C. Robertson, Effects of supplementary feeding on the offspring sex ratio of kakapo: A dilemma for the conservation of a polygynous parrot. *Biol. Conserv.* **107**, 13–18 (2002).

15. D. Raubenheimer, S. J. Simpson, The challenge of supplementary feeding: Can geometric analysis help save the kakapo? *Notornis* **53**, 100–111 (2006).

16. H. Liu, H. Ma, S. M. Cheyne, S. T. Turvey, Recovery hopes for the world's rarest primate. *Science* **368**, 1074–1074 (2020).

17. W. Wu, X. M. Wang, F. Claro, D. Youzhong, A. C. Souris, C. D. Wang, C. H. Wang, R. Berzins, The current status of the Hainan black-crested gibbon *Nomascus sp. cf. nasutus hainanus* in Bawangling National Nature Reserve, Hainan, China. *Oryx* **38**, 452–456 (2004).

18. J. Zhou, F. Wei, M. Li, J. F. Zhang, D. Wang, R. L. Pan, Hainan black-crested gibbon is headed for extinction. *Int. J. Primatol.* **26**, 453–465 (2005).

19. Z. Liu, Y. Zhang, H. Jiang, S. Charles, Population structure of *Hylobates concolor* in Bawanglin Nature Reserve, Hainan, China. *Am. J. Primatol.* **19**, 247–254 (1989).

20. S. T. Turvey, E. Y. X. Lau, C. Duncan, H. Ma, H. Liu, Assessing the information-content of messy data to reconstruct population recovery dynamics for the world's rarest primate. *Ecol. Evol.* **14**, e70089 (2024).

21. T. Geissmann, B. Chan, The Hainan black crested gibbon: Most critically endangered ape. *Folia Primatol.* **75**, 116–116 (2004).

22. B. P. L. Chan, J. R. Fellowes, T. Geissmann, J. Zhang, "Hainan Gibbon Status Survey and Conservation Action Plan" (Kadoorie Farm & Botanic Garden, 2005).

23. R. Mittermeier, J. Ratsimbazafy, A. Rylands, L. Williamson, J. Oates, D. Mbora, J. Ganzhorn, E. Rodríguez-Luna, E. Palacios, E. Heymann, M. Kierulff, Y. Long, J. Supriatna, C. Roos, S. Walker, J. Aguiar, Primates in Peril: The World's 25 Most Endangered Primates 2006–2008. *Primate Conserv.* **22**, 1–40 (2007).

24. J. R. Fellowes, C. Bosco, P.-k. P. Lok, Z. Jiang, S. Chen, S. Yang, N. S. Chit, B. Garden, Current status of the Hainan gibbon (*Nomascus hainanus*): Progress of population monitoring and other priority actions. *Asian Primates J.* **1**, 2–11 (2008).

25. National Park of Hainan Tropical Rainforest, "*Nomascus hainanus*" (China National Parks, 2022); https://www.hntrnp.com/news/2022/show-1004.html [accessed 10 January 2024].

26. W. Bleisch, B. Chan, S. M. Cheyne, P. F. Fan, T. Geissmann, M. Holdgate, F. Hu, V. Martin, R. Mittermeier, J. Smart, S. T. Turvey, Y. F. Tang, A. Zhang, J. Zhou, "Hainan Gibbon Case Study: Effectively Saving a Critically Endangered Species – *Nomascus hainanus*" (IUCN, 2021).

27. Y. Wengel, L. Ma, L. Han, Will the new Chinese National Parks system save the world's rarest primate, the Hainan gibbon? *Anim. Conserv.* **27**, 141–145 (2024).

28. J. Zhou, F. Wei, M. Li, C. B. Pui Lok, D. Wang, Reproductive characters and mating behaviour of wild *Nomascus hainanus*. *Int. J. Primatol.* **29**, 1037–1046 (2008).

29. J. V. Bryant, V. A. Olson, H. J. Chatterjee, S. T. Turvey, Identifying environmental versus phylogenetic correlates of behavioural ecology in gibbons: Implications for conservation management of the world's rarest ape. *BMC Evol. Biol.* **15**, 171 (2015).

30. J. V. Bryant, X. Zeng, X. Hong, H. J. Chatterjee, S. T. Turvey, Spatiotemporal requirements of the Hainan gibbon: Does home range constrain recovery of the world's rarest ape? *Am. J. Primatol.* **79**, 1–13 (2017).

31. D. Zhang, X. Qi, S. Liu, K. Lu, Y. Chen, W. Long, Seasonal home range utilization of Hainan gibbons (*Nomascus hainanus*) in a secondary tropical forest of Hainan Island, south China. *Glob. Ecol. Conserv.* **54**, e03063 (2024).

32. X. Zhong, C. Zhu, Y. Wang, X. Qi, P. Fan, Quantified diet provides suggestions for habitat restoration for the world's rarest primate. *Biol. Conserv.* **284**, 110215 (2023).

33. S. J. Simpson, D. Raubenheimer, *The Nature of Nutrition* (Princeton Univ. Press, 2012).

34. J. V. Bryant, D. Gottelli, X. Zeng, X. Hong, B. P. L. Chan, J. R. Fellowes, Y. Zhang, J. Luo, C. Durrant, T. Geissmann, H. J. Chatterjee, S. T. Turvey, Assessing current genetic status of the Hainan gibbon using historical and demographic baselines: Implications for conservation management of species of extreme rarity. *Mol. Ecol.* **25**, 3540–3556 (2016).

35. Y. Guo, J. Chang, L. Han, T. Liu, G. Li, P. A. Garber, N. Xiao, J. Zhou, The genetic status of the critically endangered hainan gibbon (*Nomascus hainanus*): A species moving toward extinction. *Front. Genet.* **11**, 608633 (2020).

36. M. E. Gilpin, M. E. Soulé, Minimum viable populations: Processes of species extinction, in *Conservation biology:the science of scarcity and diversity*, M. E. Soulé, Ed. (Sinauer Associates, 1986).

37. Q. He, S. Yan, P. A. Garber, B. Ren, X. Qi, J. Zhou, Habitat restoration is the greatest challenge for population recovery of Hainan gibbons (*Nomascus hainanus*). *Integr. Zool.* **18**, 630–646 (2023).

38. X. Liu, Rediscovery of the Hainan gibbon and identification of its scientific name. *Chin. J. Zool.* **13**, 26–28 (1978).

39. L. H. Xu, Z. H. Liu, W. P. Liao, X. H. lI, S. M. Yu, J. C. Qiu, Y. Y. Zhou, J. X. Deng, G. X. Guan, J. Z. Lu, K. Yan, Birds and animals of Hainan Island (Science Press, Beijing, China, 1983), pp. 315–316.

40. National Research Council, Chapter 2: Energy, in *Nutrient Requirements of Nonhuman Primates* (The National Academies, ed. 2, 2003).

41. N. Q. Hu, Z. H. Guan, B. Huang, W. H. Ning, K. He, P. F. Fan, X. L. Jiang, Dispersal and female philopatry in a long-term, stable, polygynous gibbon population: Evidence from 16 years field observation and genetics. *Am. J. Primatol.* **80**, e22922 (2018).

42. W. Li, “Genetic changes of hainan gibbon *Nomascus hainanus* after population expansion,” thesis, Guizhou Normal University, 2024.

43. U. H. Reichard, J. Prime, Western black crested gibbon – *Nomascus concolor*, in *All the World's Primates*. N. Rowe, M. Myewrs, Eds. (Pogonias Press, 2016), pp. 651–652.

44. H.-L. Fei, C. Thompson, P.-F. Fan, Effects of cold weather on the sleeping behavior of Skywalker hoolock gibbons (*Hoolock tianxing*) in seasonal montane forest. *Am. J. Primatol.* **81**, e23049 (2019).

45. K. Steudel, The physiology and energetics of movement: Effects on individuals and groups, in *On the Move: How and why animals travel in groups.* S. BoinskI, P. A. Garber, Eds. (The University of Chicago Press, 2000).

46. B. M. Galdikas, A. Ashbury, Reproductive parameters of female orangutans (*Pongo pygmaeus wurmbii*) 1971-2011, a 40-year study at Tanjung Puting National Park, Central Kalimantan, Indonesia. *Primates* **54**, 61–72 (2013).

47. A. Rangel-Negrín, A. Coyohua-Fuentes, D. R. Chavira-Ramírez, D. Canales-Espinosa, P. A. D. Dias, Energetic constraints on the reproduction of female mantled howlers. *Am. J. Primatol.* **80**, e22925 (2018).

48. T. Geissmann, Reassessment of age of sexual maturity in gibbons (*hylobates spp*.). *Am. J. Primatol.* **23**, 11–22 (1991).

49. B. L. Burns, D. S. Judge, The varied path to adulthood: Plasticity in developmental timing in hylobatids. *Am. J. Primatol.* **78**, 610–625 (2016).

50. J. U. Ganzhorn, Effects of introduced *Rattus rattus* on endemic small mammals in dry deciduous forest fragments of western Madagascar. *Anim. Conserv.* **6**, 147–157 (2003).

51. K. Mokross, J. R. Potts, C. L. Rutt, P. C. Stouffer, What can mixed-species flock movement tell us about the value of Amazonian secondary forests? Insights from spatial behavior. *Biotropica* **50**, 664–673 (2018).

52. P.-F. Fan, H.-S. Ai, H.-L. Fei, D. Zhang, S.-D. Yuan, Seasonal variation of diet and time budget of Eastern hoolock gibbons (*Hoolock leuconedys*) living in a northern montane forest. *Primates* **54**, 137–146 (2013).

53. P. Fan, Q. Ni, G. Sun, B. Huang, X. Jiang, Gibbons under seasonal stress: The diet of the black crested gibbon (*Nomascus concolor*) on Mt. Wuliang, Central Yunnan, China. *Primates* **50**, 37–44 (2009).

54. Z. Zhang, R. Zang, Diversity and distribution of food plants: Implications for conservation of the critically endangered Hainan gibbon. *Nat. Conserv. Bulgaria* **31**, 17–33 (2018).

55. Y. Guo, D. Peng, L. Han, T. Liu, G. Li, P. A. Garber, J. Zhou, Mitochondrial DNA control region sequencing of the critically endangered Hainan gibbon (*Nomascus hainanus*) reveals two female origins and extremely low genetic diversity. *Mitochondrial DNA B. Resour.* **6**, 1355–1359 (2021).

56. M. Kardos, Y. L. Zhang, K. M. Parsons, A. Yunga, H. Kang, X. Xu, X. Liu, C. O. Matkin, P. J. Zhang, E. J. Ward, M. B. Hanson, C. Emmons, M. J. Ford, G. Y. Fan, S. H. Li, Inbreeding depression explains killer whale population dynamics. *Nat. Ecol. Evol.* **7**, 675–686 (2023).

57. M. Zhang, J. R. Fellowes, X. Jiang, W. Wang, B. P. L. Chan, G. Ren, J. Zhu, Degradation of tropical forest in Hainan, China, 1991–2008: Conservation implications for Hainan Gibbon (*Nomascus hainanus*). *Biol. Conserv.* **143**, 1397–1404 (2010).

58. B. P. L. Chan, Y. F. P. Lo, X. J. Hong, C. F. Mak, Z. Ma, First use of artificial canopy bridge by the world's most critically endangered primate the Hainan gibbon *Nomascus hainanus*. *Sci. Rep.* **10**, 15176 (2020).

59. Y. Zhang, P. D. Mathewson, W. P. Porter, Q. Zhang, Mechanistically simulating the effects of climate change to identify conservation hotspots and reproduction potential for an endangered species. *Biol. Conserv.* **302**, 110905 (2025).

60. G. M. Mace, N. J. Collar, K. J. Gaston, C. Hilton-Taylor, H. R. Akcakaya, N. Leader-Williams, E. J. Milner-Gulland, S. N. Stuart, Quantification of extinction risk: IUCN's system for classifying threatened species. *Conserv. Biol.* **22**, 1424–1442 (2008).

61. A. Dobson, A. Lyles, Black-footed ferret recovery. *Science* **288**, 985, 988 (2000).

62. L. Williamson, A. Feistner, Habituating primates: Process, techniques, variables and ethics, in *Field and Laboratory Methods in Primatology: A Practical Guide*, J. Setchell, A. Feistner, Eds. (Cambridge Univ. Press, ed. 2, 2011), pp. 33–50.

63. J. Altmann, Observational study of behavior: Sampling methods. *Behaviour* **49**, 227–266 (1974).

64. P.-F. Fan, H.-L. Fei, C.-Y. Ma, Behavioral responses of Cao Vit Gibbon (*Nomascus Nasutus*) to variations in food abundance and temperature in Bangliang, Jingxi, China. *Am. J. Primatol.* **74**, 632–641 (2012).

65. H. Fei, M. de Guinea, L. Yang, C. A. Chapman, P. Fan, Where to sleep next? Evidence for spatial memory associated with sleeping sites in Skywalker gibbons (*Hoolock tianxing*). *Anim. Cogn.* **25**, 891–903 (2022).

66. B. Kranstauber, M. Smolla, A. K. Scharf, Move: Visualizing and analyzing animal track data. R package version 4.2.6 (2024); https://CRAN.R-project.org/package=move.

67. J. M. Rothman, D. Raubenheimer, C. A. Chapman, Nutritional geometry: Gorillas prioritize non-protein energy while consuming surplus protein. *Biol. Lett.* **7**, 847–849 (2011).

68. P. J. Van Soest, J. B. Robertson, B. A. Lewis, Methods for dietary fiber, neutral detergent fiber, and nonstarch polysaccharides in relation to animal nutrition. *J. Dairy Sci.* **74**, 3583–3597 (1991).

69. J. M. Rothman, C. A. Chapman, A. N. Pell, Fiber-bound nitrogen in gorilla diets: Implications for estimating dietary protein intake of primates. *Am. J. Primatol.* **70**, 690–694 (2008).

70. J. M. Rothman, C. A. Chapman, P. J. Van Soest, Methods in primate nutritional ecology: A User's guide. *Int. J. Primatol.* **33**, 542–566 (2012).

71. R. Hou, C. A. Chapman, O. Jay, S. Guo, B. Li, D. Raubenheimer, Cold and hungry: Combined effects of low temperature and resource scarcity on an edge-of-range temperate primate, the golden snub-nose monkey. *Ecography* **43**, 1672–1682 (2020).

72. S.-M. Gao, H.-L. Fei, Q. Li, L.-Y. Lan, L.-N. Huang, P.-F. Fan, Eco-evolutionary dynamics of gut phageome in wild gibbons (*Hoolock tianxing*) with seasonal diet variations. *Nat. Commun.* **15**, 1254–1254 (2024).

73. N. Hon, A. M. Behie, J. M. Rothman, K. G. Ryan, Nutritional composition of the diet of the northern yellow-cheeked crested gibbon (*Nomascus annamensis*) in northeastern Cambodia. *Primates* **59**, 339–346 (2018).

74. M. Kleiber, *The Fire of Life: An Introduction to Animal Energetics* (Wiley, 1961).

75. C. R. Taylor, K. Schmidt-Nielsen, J. L. Raab, Scaling of energetic cost of running to body size in mammals. *Am. J. Physiol.* **219**, 1104–1107 (1970).

76. C. Key, C. Ross, Sex differences in energy expenditure in non-human primates. *Proc. R. Soc. B. Biol. Sci.* **266**, 2479–2485 (1999).

77. W. R. Leonard, M. L. Robertson, Comparative primate energetics and hominid evolution. *Am. J. Phys. Anthropol.* **102**, 265–281 (1997).

78. H. G. Ma, C. Y. Ma, H. L. Fei, L. Yang, P. F. Fan, Cao Vit Gibbons (*Nomascus nasutus*) sing at higher elevation but not in peripheral areas of their home range in a karst forest. *Int. J. Primatol.* **41**, 701–713 (2020).

79. O. W. Portman, Nutritional requirements of nonhuman primates, in *Feeding and Nutrition of Nonhuman Primates*, R. S. Harris, Ed. (Academic Press, 1970), pp. 87–115.

80. H. Deng, M. Zhang, J. Zhou, Recovery of the critically endangered hainan gibbon *Nomascus hainanus*. *Oryx* **51**, 161–165 (2017).

81. P. L. Fan, X. He, Y. Z. Yang, X. F. Liu, H. B. Zhang, L. Yuan, W. Chen, D. Z. Liu, P. F. Fan, Reproductive parameters of captive female northern white-cheeked (*Nomascus leucogenys*) and yellow-cheeked (*Nomascus gabriellae*) gibbons. *Int. J. Primatol.* **42**, 49–63 (2021).

82. D. Bates, M. Mächler, B. Bolker, S. Walker, Fitting linear mixed-effects models using lme4. *J. Stat. Softw.* **67**, 1–48 (2015).

83. A. M. Nsubuga, M. M. Robbins, A. D. Roeder, P. A. Morin, C. Boesch, L. Vigilant, Factors affecting the amount of genomic DNA extracted from ape faeces and the identification of an improved sample storage method. *Mol. Ecol.* **13**, 2089–2094 (2004).

84. L. Y. Cui, B. Y. Liu, H. M. Li, Y. X. Zhu, Y. H. Zhou, C. Su, Y. P. Tian, H. T. Xu, D. Liu, X. P. Li, Y. Ma, G. S. Jiang, H. Liu, S. H. Yang, T. M. Lan, Y. C. Xu, A simple and effective method to enrich endogenous DNA from mammalian faeces. *Mol. Ecol. Resour.* **24**, e13939 (2024).

85. P. Taberlet, G. Luikart, Non-invasive genetic sampling and individual identification. *Biol. J. Linn. Soc.* **68**, 41–55 (1999).

86. P. Taberlet, Reliable genotyping of samples with very low DNA quantities using PCR. *Nucleic Acids Res.* **24**, 3189–3194 (1996).

87. P. Bolechova, K. Jecminkova, M. Hradec, T. Kott, J. Dolezalova, Sex determination in gibbons of genus *Nomascus* using non-invasive method. *Acta Veterinaria Brno* **85**, 363–366 (2016).

88. A. D. Fiore, A rapid genetic method for sex assignment in non-human primates. *Conserv. Genet.* **6**, 1053–1058 (2006).

89. P. F. Fan, K. He, X. Chen, A. Ortiz, B. Zhang, C. Zhao, Y. Q. Li, H. B. Zhang, C. Kimock, W. Z. Wang, C. Groves, S. T. Turvey, C. Roos, K. M. Helgen, X. L. Jiang, Description of a new species of hoolock gibbon (Primates: Hylobatidae) based on integrative taxonomy. *Am. J. Primatol.* **79**, e22631 (2017).

90. S. T. Kalinowski, M. L. Taper, T. C. Marshall, Revising how the computer program CERVUS accommodates genotyping error increases success in paternity assignment. *Mol. Ecol.* **16**, 1099–1106 (2007).

91. C. Van Oosterhout, W. F. Hutchinson, D. P. M. Wills, P. Shipley, MICRO-CHECKER: Software for identifying and correcting genotyping errors in microsatellite data. *Mol. Ecol. Notes* **4**, 535–538 (2004).

92. R. Peakall, P. E. Smouse, GenAlEx 6.5: Genetic analysis in Excel. Population genetic software for teaching and research-an update. *Bioinformatics* **28**, 2537–2539 (2012).

93. L. Excoffier, H. E. Lischer, Arlequin suite ver 3.5: A new series of programs to perform population genetics analyses under Linux and Windows. *Mol. Ecol. Resour.* **10**, 564–567 (2010).

94. W. R. Rice, Analyzing tables of statistical tests. *Evolution* **43**, 223–225 (1989).

95. S. Kumar, G. Stecher, K. Tamura, MEGA7: Molecular evolutionary genetics analysis version 7.0 for bigger datasets. *Mol. Biol. Evol.* **33**, 1870–1874 (2016).

96. J. Rozas, A. Ferrer-Mata, J. C. Sánchez-DelBarrio, S. Guirao-Rico, P. Librado, S. E. Ramos-Onsins, A. Sánchez-Gracia, DnaSP 6: DNA sequence polymorphism analysis of large data sets. *Mol. Biol. Evol.* **34**, 3299–3302 (2017).

97. J. Wang, Estimating pairwise relatedness in a small sample of individuals. *Heredity (Edinb)* **119**, 302–313 (2017).

98. J. L. Wang, An estimator for pairwise relatedness using molecular markers. *Genetics* **160**, 1203–1215 (2002).

99. J. Wang, COANCESTRY: A program for simulating, estimating and analysing relatedness and inbreeding coefficients. *Mol. Ecol. Resour.* **11**, 141–145 (2011).

100. D. J. Chivers, M. V. Anandam, C. P. Grove, S. Molur, B. M. Rawson, M. C. Richardson, C. Roos, D. Whittaker, Family Hylobatidae (gibbons), in *Handbook of the Mammals of the*

World, Primates. R. A. Mittermeier, A. B. Rylands, D. E. Wilson, Eds. (Lynx Edicions, 2013), vol. 3.

101. R. L. Tilson, Family formation strategies of Kloss's gibbons. *Folia Primatol. (Basel)* **35**, 259–287 (1981).

102. J. C. Mitani, Demography of agile gibbons (*Hylobates agilis*). *Int. J. Primatol.* **11**, 411–424 (1990).

103. T. G. O'Brien, M. F. Kinnaird, Demography of Agile Gibbons (*Hylobates agilis*) in a lowland tropical rain forest of Southern Sumatra, Indonesia: Problems in paradise. *Int. J. Primatol.* **32**, 1203–1217 (2011).

104. U. H. Reichard, C. Barelli, Life history and reproductive strategies of Khao Yai *Hylobates lar*: Implications for social evolution in apes. *Int. J. Primatol.* **29**, 823–844 (2008).

105. W. Y. Brockelman, U. Reichard, U. Treescuon, J. J. Raemaekers, Dispersal, pair formation and social structure in gibbons (*Hylobates lar*). *Behav. Ecol. Sociobiol.* **42**, 329–339 (1998).

106. Y. Yi, C. Fichtel, S. Ham, H. Jang, J. C. Choe, Fighting for what it's worth: Participation and outcome of inter-group encounters in a pair-living primate, the Javan gibbon (*Hylobates moloch*). *Behav. Ecol. Sociobiol.* **74**, 96 (2020).

107. A. Choi, Y. Yi, A. Mardiastuti, J. C. Choe, Intra-group competition and social dynamics regarding dispersal and maturation in wild Javan gibbon (*Hylobates moloch*). *Sci. Rep.* **13**, 8285 (2023).

108. T. G. O'Brien, M. F. Kinnaird, A. Nurcahyo, M. Prasetyaningrum, M. Iqbal, Fire, demography and the persistence of siamang (*Symphalangus syndactylus*: Hylobatidae) in a Sumatran rainforest. *Anim. Conserv.* **6**, 115–121 (2003).

109. S. Lappan, Male care of infants in a siamang (*Symphalangus syndactylus*) population including socially monogamous and polyandrous groups. *Behav. Ecol. Sociobiol.* **62**, 1307–1317 (2008).
